# A Myosin Light Chain Is Critical for Fungal Growth Robustness in Candida albicans

**DOI:** 10.1128/mBio.02528-21

**Published:** 2021-10-05

**Authors:** Charles Puerner, Antonio Serrano, Rohan S. Wakade, Martine Bassilana, Robert A. Arkowitz

**Affiliations:** a Université Côte d’Azur, CNRS, INSERM, Institute of Biology Valrose (iBV), Parc Valrose, Nice, France; Duke University

**Keywords:** Spitzenkörper, filamentous growth, morphology, secretion

## Abstract

In a number of elongated cells, such as fungal hyphae, a vesicle cluster is observed at the growing tip. This cluster, called a Spitzenkörper, has been suggested to act as a vesicle supply center, yet analysis of its function is challenging, as a majority of components identified thus far are essential for growth. Here, we probe the function of the Spitzenkörper in the human fungal pathogen Candida albicans, using genetics and synthetic physical interactions (SPI). We show that the C. albicans Spitzenkörper is comprised principally of secretory vesicles. Mutant strains lacking the Spitzenkörper component myosin light chain 1 (Mlc1) or having a SPI between Mlc1 and either another Spitzenkörper component, the Rab GTPase Sec4, or prenylated green fluorescent protein (GFP), are viable and still exhibit a Spitzenkörper during filamentous growth. Strikingly, all of these mutants formed filaments with increased diameters and extension rates, indicating that Mlc1 negatively regulates myosin V, Myo2, activity. The results of our quantitative studies reveal a strong correlation between filament diameter and extension rate, which is consistent with the vesicle supply center model for fungal tip growth. Together, our results indicate that the Spitzenkörper protein Mlc1 is important for growth robustness and reveal a critical link between filament morphology and extension rate.

## INTRODUCTION

In virtually all filamentous fungi, an electron-dense vesicle cluster at the filament tips called a Spitzenkörper has been observed (1–3) and has been proposed to regulate hyphal growth and function as a vesicle supply center in a range of fungi (4–6). This model was obtained from a computer simulation of fungal morphogenesis with the following assumptions (5). (i) Cell surface expansion is derived from tip targeted vesicles. (ii) Vesicles are released from a vesicle supply center (VSC) (modeled as an idealized point source within the cell). (iii) Vesicles are released from the VSC to the surface in a random direction. The model predicts that the shape of the hypha is generated by linear displacement of the VSC described by the hyphoid equation


(1) $$y=x\cot\frac{V\cdot x}{N}$$

where *N* is the number of vesicles released from the VSC per unit time, *V* is the rate of linear displacement of the VSC, and $\frac{N}{V}$ is the distance between the apical wall and the VSC. Support for this model predicting hyphal shape came with early studies of the plant fungal pathogen Rhizoctonia solani, in which morphology was followed upon disturbing growth. Specifically, dislocation of the Spitzenkörper, i.e., an increase in $\frac{N}{V}$ led to an increase in tip diameter (4). Furthermore, the hyphal tips of 32 fungal species were shown to approximate the hyphoid shape as described in equation 1 (7). Indeed, the position of the Spitzenkörper appears to anticipate a change in growth direction in Aspergillus niger and R. solani (2, 7). When grown on surfaces, Candida albicans hyphal tips, as well as the Spitzenkörper, are asymmetrically positioned toward the substrate, and the position of this structure, which moves upon filament contact, was not an absolute predictor of growth direction (8). The studies of A. niger and R. solani have also analyzed the consequences of altering the position of the Spitzenkörper $\left(\frac{N}{V}\right)$ with respect to filament morphology (2, 7). From the hyphoid equation (equation 1), the maximum diameter of the hyphae was derived as follows:


(2) $$D=2\pi \frac{N}{V}$$

where *D* is the maximum hyphal diameter. This equation predicts that VSC links the diameter of the hyphae to its extension rate. As $\frac{N}{V}$ is the position of the vesicle supply center relative to the apical wall, this indicates that the hyphal diameter is proportional to the position of the vesicle supply center, i.e., the closer to the apical wall, the narrower the hyphal diameter and vice versa. Similarly, as the number of vesicles released from the vesicle supply center per unit time increases, i.e., increased extension rate, the hyphal diameter should increase, assuming the position of the vesicle supply center relative to the wall $\left(\frac{N}{V}\right)$ does not change substantially.

In filamentous fungi, a number of proteins have been localized to the Spitzenkörper, including Rab GTPases (Rab11 and Rab8 homologs, as well as Rab6 and Rab1 in Aspergillus nidulans) (9–16), Rab guanine nucleotide exchange factors (GEFs) (e.g., Sec2) (17), the polarisome protein Spa2, the nuclear dbf2-related (NDR) kinase COT-1 (18), lipid flippases (19, 20), the myosin V motor (21, 22) and myosin light chain (9, 23), the glycolysis enzyme GPI-1 (24), the coiled-coiled protein SPZ-1 (24), scaffold proteins (Leashin-2 and Janus-1) (24), chitin and glucan synthases (14, 25–31) and the formin Bni1 (23, 32, 33). In C. albicans, the Spitzenkörper has been visualized by fluorescence microscopy (10, 15, 17, 23, 33), and electron microscopy revealed that it is comprised of a homogeneous vesicle population of approximately 60 vesicles (16), in contrast to Neurospora crassa, where a layered structure of micro- and macrovesicles is observed (31). N. crassa Spitzenkörper mutants with altered composition of this structure (*Δspz-1*, *Δjns-1*, *Δspa-2*, and *Δmyo-5*) exhibit reduced extension rates (24). In C. albicans, perturbation of actin cables (10, 16, 23) or secretion in a *sec3* mutant disrupted the Spitzenkörper (16, 34). Mutation of the C. albicans Ras-like GTPase Rsr1 also resulted in the position of the Spitzenkörper to meander during growth (8). Finally, two polarisome components, Spa2 and Bud6, have been shown to be required for the integrity of the C. albicans Spitzenkörper, and deletion of either component resulted in wider and less polarized filaments (23).

Here, we used mutants of a myosin light chain, Mlc1, which localizes to the Spitzenkörper, to probe the function of this structure using a quantitative analysis of C. albicans hyphal growth and morphology. We first demonstrated that, in the absence of Mlc1, filamentous growth still occurs and a Spitzenkörper is present, yet *mlc1* deletion mutants exhibited wider filaments with increased extension rates. In addition to this deletion mutant, we generated two strains with constitutive synthetic physical interactions (SPI) (35) between Mlc1 and either the Rab8 GTPase Sec4 or prenylated green fluorescent protein (GFP). Strikingly, with these mutants, similar to the *mlc1* deletion mutant, we observed a strong correlation between filament diameter and extension rate, as predicted by the vesicle supply center model. Together, our results reveal that the Spitzenkörper protein Mlc1 is essential for growth robustness.

## RESULTS

**FIG 1:**
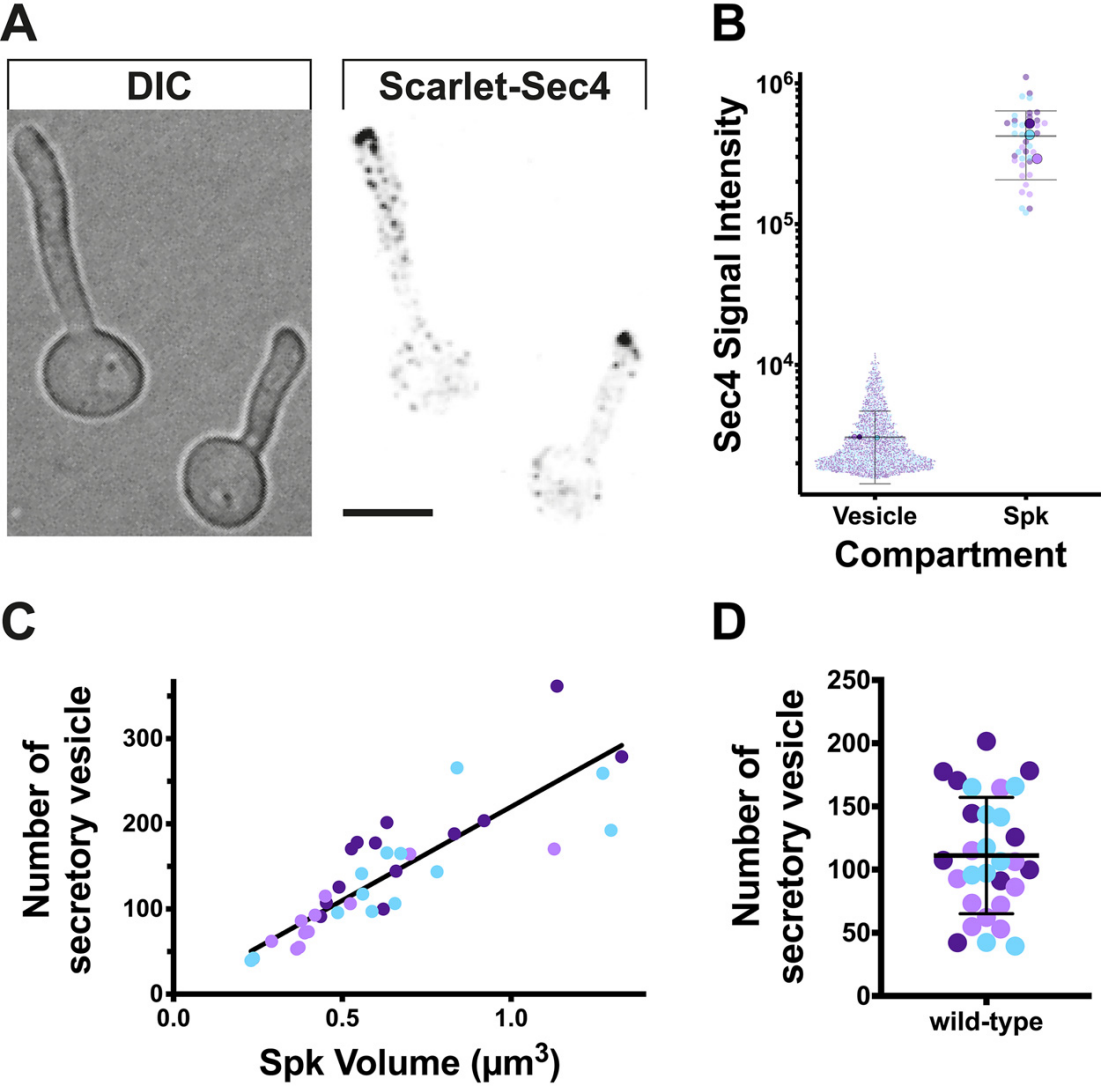
The Spitzenkörper is comprised essentially of secretory vesicles. (A) Sec4-labeled secretory vesicles and Spitzenkörper. A strain expressing Scarlet-Sec4 as the sole copy (PY4554), was incubated with FCS on agarose pads at 37°C for 1 h, imaged (31 × 0.2 μm z-sections). DIC, differential interference contrast microscopy. Bar, 5 μm. (B) Sec4 signal intensity of secretory vesicles and Spitzenkörper (Spk). Fluorescence signals (5.5 standard deviations above the mean signal) were identified from images in panel A with the smallest (2 to 8 voxels; 0.0068 to 0.027 μm^3^) classified as vesicles and the largest (68 to 394 voxels; 0.23 to 1.33 μm^3^) classified as Spitzenkörpers. The different colors depict the results of three independent agarose pad experiments (*n *= 38 cells) with larger symbols representing the means for each pad and horizontal lines indicating means and bars indicating standard deviations. (C) The number of secretory vesicles in the Spitzenkörper correlates with its volume. The mean signal per secretory vesicle (3,059 ± 1,630) was used to determine the number of secretory vesicles in the Spitzenkörper. A linear regression with the *y*-intercept constrained to zero yielded an *r*^2^ value of 0.71. The determination of the number of secretory vesicles in the Spitzenkörper assumes that all the vesicles contain this Rab GTPase. (D) The Spitzenkörper is comprised of approximately 100 secretory vesicles. The mean number of secretory vesicles in Spitzenkörpers that ranged from 0.29 to 0.98 μm^3^ (80% of the cells) was 110 ± 46. The mean (horizontal line) and standard deviation (error bar) are shown.

First, we set out to examine the composition of the C. albicans Spitzenkörper, specifically if it was predominantly composed of secretory vesicles. Using a functional Scarlet-Sec4 fusion, present as the sole copy, we quantitated the signal in the smallest structures (ranging in size between 0.0068 and 0.027 μm^3^) and compared it to the signal associated with the Spitzenkörper (Fig. 1A and B). We used the red fluorescent protein mScarlet, as there was less background signal in the cells compared to using GFP, which was critical for identifying individual secretory vesicles. The mean signal per secretory vesicle (3,059 ± 1,630) was then used to determine the number of secretory vesicles in the Spitzenkörper, and we observed a good correlation between this number of secretory vesicles and the volume of the Spitzenkörper (Fig. 1C). The average number of secretory vesicles in the Spitzenkörper of 110 ± 46 (Fig. 1D) is in good agreement with the number of total vesicles determined by electron microscopy of 60 to 75 (16). Hence, these results are consistent with the notion that the Spitzenkörper is comprised essentially of secretory vesicles. While we did observe some variation in the absolute number of secretory vesicles in the Spitzenkörper, the intensity of the Sec4 signal at this structure was constant over time, suggesting that the rate of vesicles arriving equals the rate of vesicles departing from it.

**FIG 2:**
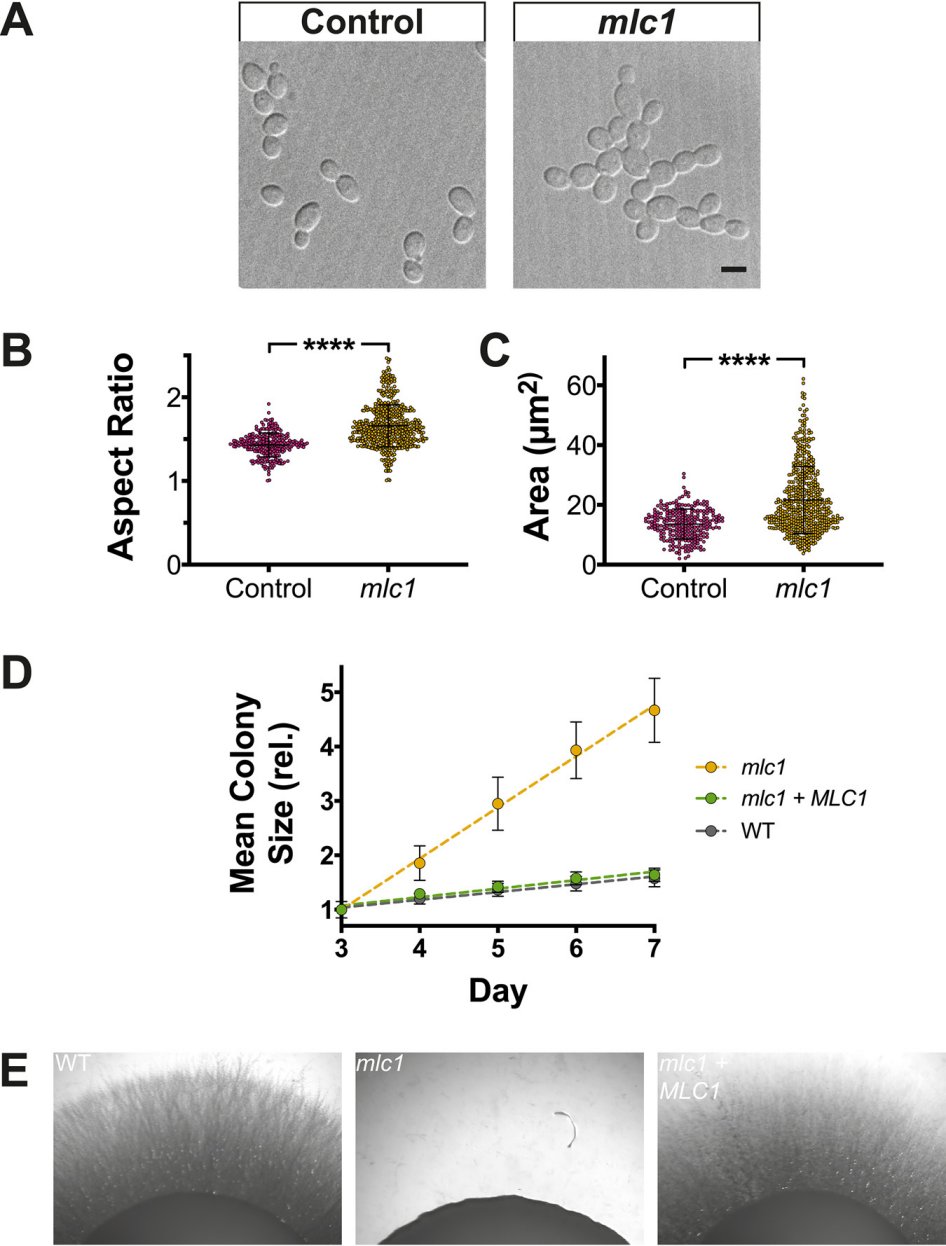
In the absence of Mlc1, cells are enlarged and grow faster during budding growth yet are defective for invasive growth. (A to C) Deletion of results in larger budding cells. DIC images of *mlc1Δ*/*MLC1* (Control) and *mlc1Δ*/*mlc1Δ* (*mlc1*) cells expressing Scarlet-Sec4 and either Nop1-GFP (PY5716) or Cdc10-GFP (PY5720), respectively, are shown. Bar, 5 μm. (B and C) The aspect ratios (long axis over short axis) and area (assuming a uniform ellipse) were determined (*n *= 250 to 500 cells) with **** indicating *P* value of <0.0001. (D) Colonies of the *mlc1* mutant grow faster than the wild type. The indicated strains, including wild-type (WT; PY4860), *mlc1* (*mlc1Δ*/*mlc1Δ*; PY4754), and *mlc1 *+* MLC1* (*mlc1Δ*/*mlc1Δ* + *MLC1*; PY5658) were grown on rich medium at 30°C, and colony diameter (*n *= 25 to 50) was determined after 3 to 7 days incubation. Symbols are mean values of the normalized colony size at 3 days, with error bars indicating standard deviations; the lines are best fits with *r*^2^ > 0.94. (E) Mlc1 is required for invasive growth. Indicated strains, expressing Scarlet-Sec4 (WT, PY4860; *mlc1*, PY5451; *mlc1 + MLC1*, PY5661) were spotted on rich medium containing serum and incubated at 37°C for 7 days.

**FIG 3:**
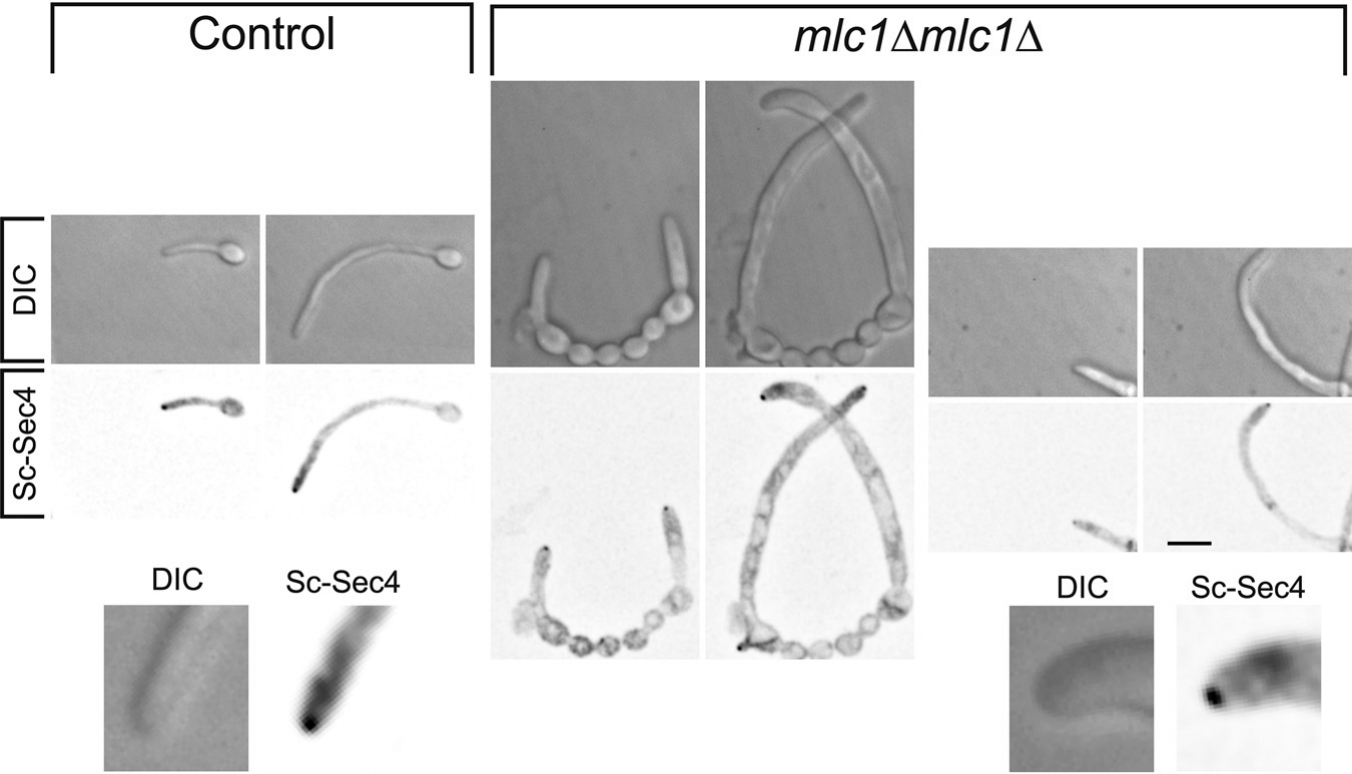
The myosin light chain 1 is not required for filamentous growth or Spitzenkörper formation. Indicated strains (control; *mlc1Δ*/*MLC1* [PY5018] and *mlc1* [*mlc1Δ*/*mlc1Δ*, PY5451]) expressing Scarlet-Sec4 were incubated and imaged as described in the legend to Fig. 1A, and sum projections are shown with the second image in each panel 1 h after the first image.

**FIG 4:**
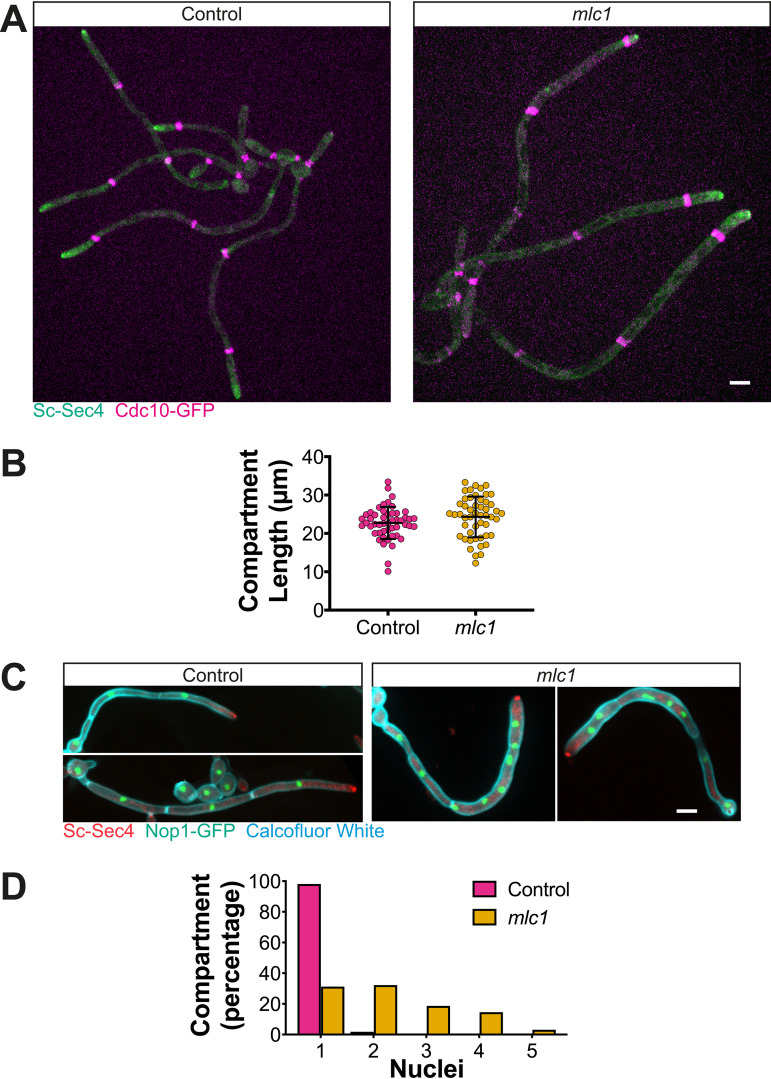
Filament compartment length is unaffected in the *mlc1* mutant, which is multinucleate. (A) Mlc1 is not required for septin distribution. Fluorescent images of strains *mlc1Δ*/*MLC1* (Control; PY5713) and *mlc1Δ*/*mlc1Δ* (*mlc1*; PY5717) cells expressing Scarlet-Sec4 and Cdc10-GFP are shown. Images are maximum projections of 26 × 0.4 μm z-sections of cells grown as described in the legend to Fig. 1 for 3 h. (B) The filament compartment length is unaffected in *mlc1* cells. The length between septin bands was measured (*n *= 50 compartments, 25 to 30 filaments) in filaments of cells in panel A, incubated on pads between 1 and 3 h. Values are means ± standard deviations (error bars) with no significant difference between Control and *mlc1* compartment length. (C) Multiple nuclei are observed in the *mlc1* mutant. The indicated cells expressing Scarlet-Sec4 and Nop1-GFP (WT, PY5716; *mlc1*, PY5720) were incubated with serum at 37°C for 3 h and stained with Calcofluor white to reveal septa. Images are maximum projections of 26 × 0.4 μm z-sections. (D) The majority of *mlc1* filament compartments are multinucleate. The number of nuclei was quantitated from the images of cells from panel C (*n *= 100 compartments, 30 to 70 filaments). Bars, 5 μm.

**FIG 5:**
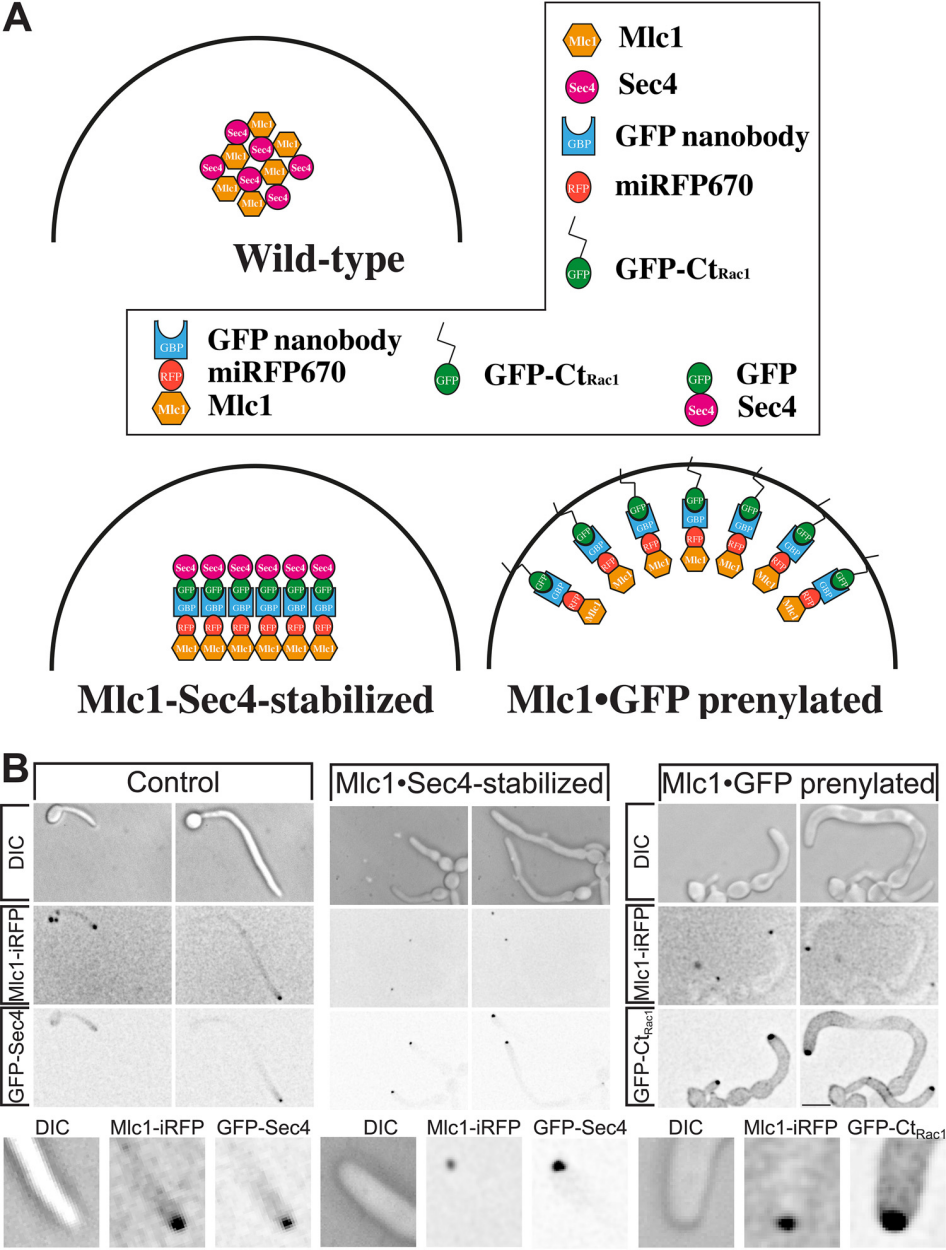
Synthetic interaction of Mlc1 with either Sec4 or prenylated GFP perturbs filamentous growth. (A) Schematic showing plasma membrane targeting of Mlc1 or stabilization with Sec4. The different domains and fusion proteins are indicated in the box. (B) Altering the stability or distribution of Mlc1 at the Spitzenkörper perturbs filamentous growth. Wild-type cells expressing Mlc1-iRFP and GFP·Sec4 (Control; PY4809), Mlc1-iRFP-GNB (iRFP stands for near-*i*nfra*r*ed *f*luorescent *p*rotein and GNB for GFP nanobody) and GFP-Sec4 (Mlc1·Sec4-stablilized; PY5405) or Mlc1-iRFP-GNB and GFP-Ct_Rac1_ (Mlc1·GFP prenylated; PY5409) were imaged as described in the legend to Fig. 2A. Note that in the absence of Mlc1-iRFP-GNB, GFP-Ct_Rac1_ (Rac1 carboxy terminus) is observed uniformly on the plasma membrane (62, 63) (Fig. 7A).

Investigating the function of the Spitzenkörper has been hampered as central components are required for viability. For example, in C. albicans, the Spitzenkörper components Sec4 (10) and its GEF Sec2 (17), as well as the Rab11 homolog Ypt31 (15) (see Fig. S1A in the supplemental material), are essential. One Spitzenkörper component that is not essential for viability in C. albicans is the formin Bni1, which is partially redundant with Bnr1 (33, 36). We examined filamentous growth in a *bni1* deletion mutant, and Fig. S2 shows that *bni1* filaments were wider, yet their extension rate was substantially reduced. However, we did not observe a cluster of secretory vesicles in this mutant, using Scarlet-Sec4 (Fig. S2D), consistent with data on other fungi (24) that in the absence of a Spitzenkörper, filamentous growth is reduced. In N. crassa, mutants lacking the polarisome component SPA-2, the scaffolding proteins Janus-1 (JNS-1) and Leashin-2 (LAH-2), as well as the coiled-coil protein SPZ-1 have a reduced growth rate, with mutants lacking the myosin V motor protein exhibiting very little growth (24). Similarly, in A. nidulans, myosin V mutants are wider and extend slower (12, 22, 37). To probe the function of the C. albicans Spitzenkörper, we generated mutants of the myosin light chain 1 (Mlc1). Homozygous *mlc1* deletion mutants were viable, with a doubling time comparable to that of the wild-type strain during budding growth, albeit with chains of cells that suggest a cytokinesis defect (Fig. 2A). Of note, it was shown that the myosin V homolog, Myo2, is also not essential for viability in C. albicans (38). The *mlc1* cells were slightly elongated and somewhat larger than control cells (Fig. 2B and C). Colonies of the *mlc1* mutant grew faster than the wild-type and complemented strains on rich medium, yet this mutant was unable to form invasive filaments in serum-containing agar medium (Fig. 2D and E). The *mlc1* mutant was nonetheless able to form filaments that resembled hyphae in liquid serum medium (Fig. 3), with lengths of the filament compartments identical to those of the control cells (Fig. 4A and B) and a tip-localized cluster of Sec4 also similar to control cells (Fig. 3 and Fig. 4A and C). However, we observed on average two nuclei per filament compartment in the *mlc1* mutant filaments, compared to a single nucleus in the control cells (Fig. 4C and D). To further probe Mlc1 function at the Spitzenkörper, we generated two mutants, each with a specific synthetic physical interaction (SPI). We sought to determine whether linking Mlc1 to prenylated GFP, which localizes to the plasma membrane, would alter the Spitzenkörper and perturb filament growth. We predicted that broadening the distribution of the Spitzenkörper should result in wider filaments that, according to the VSC model, should also extend faster. In Saccharomyces cerevisiae, Mlc1 has been shown to form a complex with Myo2 and/or Sec4 on secretory vesicles (39), and Myo2 has been shown to bind Sec4 (40). Given that Mlc1, Myo2, and Sec4 are likely to function together also in C. albicans, we examined whether stabilizing this interaction might increase the number of vesicles targeted to the site of fusion at the plasma membrane, thereby accelerating filament extension. To generate a Mlc1·GFP prenylated mutant, one copy of Mlc1 was fused to a GFP nanobody and expressed together with prenylated GFP (Fig. 5A). In contrast, to generate a Mlc1·Sec4-stabilized mutant, the interaction between Mlc1 and Sec4 was stabilized, using a GFP nanobody fused to one copy of Mlc1 in cells expressing GFP-Sec4 (Fig. 5A). Both of these SPI mutants were viable and formed filaments that appeared larger than the control cells, in serum inducing medium (Fig. 5B). In both mutants, a cluster of Sec4 was observed at the filament tip (Fig. 5B). Furthermore, Ypt31 also localized to a cluster at the tip of *mlc1* mutant filaments (Fig. S1B), which confirms that a Spitzenkörper still forms when Mlc1 is altered.

10.1128/mBio.02528-21.1 FIG S1 *YPT31* is essential for viability and localizes to the Spitzenkörper in the *mlc1* mutant. Exponentially growing cells of the indicated strains (WT, PY173; *ypt31*Δ/*pTetYPT31*, PY2896; *ypt31*Δ/*pTetYPT31 *+* Ch-YPT31*, PY3455) were spotted on YEPD medium in the presence or absence of doxycycline (Dox) and imaged after 2 days incubation at 30°C. The *mlc1* mutant (*mlc1Δ*/*mlc1Δ*) expressing mCh-Ypt31 (PY5020) was grown and imaged as described in the legend to Fig. 1, and images are maximum projections of 26 × 0.4 μm z-sections every 10 min with two examples shown. Bar, 5 μm. Download FIG S1, PDF file, 0.5 MB. Copyright © 2021 Puerner et al. 2021 Puerner et al. https://creativecommons.org/licenses/by/4.0/ This content is distributed under the terms of the Creative Commons Attribution 4.0 International license.

10.1128/mBio.02528-21.2 FIG S2 In the absence of the formin Bni1, filaments are wider but do not extend faster. (A) Bni1 is critical for maintaining filament diameter. Filament diameters of wild-type control (PY5018) and *bni1* mutant (*bni1Δ*/*bni1Δ*; PY5435) cells were determined from time-lapse experiments as described in the legend to Fig. 8. Extension rates were normalized to the wild-type strain. Bars indicate means and standard deviations (*n *= 45 to 50 cells) with **** indicating a *P* value of <0.0001. (B) Bni1 is important for filament extension rate. The extension rates of the indicated strains were determined as described in the legend to Fig. 8A. (C) A *bni1* mutant filament volume increases over time compared to that of the wild type. Rates of volume change were calculated from measurements in panels A and B, using cross-sectional area and extension rate and normalized to the means of the respective control strain. (D) In the absence of Bni1, a cluster of secretory vesicles is not observed at the filament tip. A *bni1* mutant (*bni1Δ*/*bni1Δ*) strain expressing Scarlet-Sec4 (PY5435) was grown and imaged as described in the legend to Fig. 1; maximum projections of 21 × 0.4 μm z-sections every 10 min are shown. Bar, 5 μm. Download FIG S2, PDF file, 0.3 MB. Copyright © 2021 Puerner et al. 2021 Puerner et al. https://creativecommons.org/licenses/by/4.0/ This content is distributed under the terms of the Creative Commons Attribution 4.0 International license.

**FIG 6:**
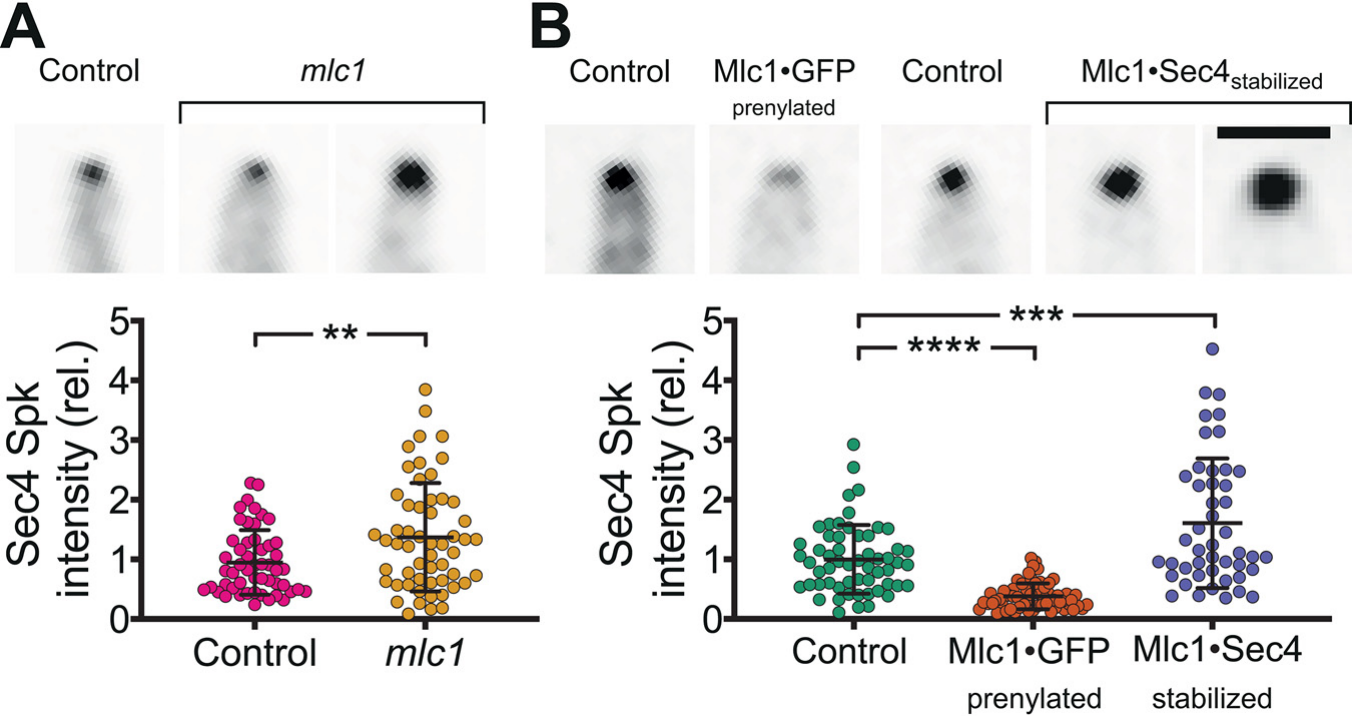
Altering the amount, distribution, and stability of Mlc1 at the Spitzenkörper. (A) Mlc1 is required for regulating the number of secretory vesicles. Indicated strains *mlc1Δ*/*MLC1* (Control; PY5018) and *mlc1Δ*/*mlc1Δ* (*mlc1*; PY5451), expressing Scarlet-Sec4, were imaged after incubation with FCS at 37°C for 1 h with images showing sum projection of filament tips (the two examples for *mlc1* illustrate the variation of Spitzenkörper signals). Vesicle clusters were identified in sum projections by signal intensities 8 standard deviations above the mean. The intensity values were normalized to the mean of the control strain. Means (horizontal lines) and standard deviations (error bars) (*n *= 57) are shown, with ** indicating a *P* value of <0.005. rel., relative. (B) Stabilization of Mlc1-Sec4 interaction results in an increase in secretory vesicles at the Spitzenkörper. Indicated strains, control expressing Scarlet-Sec4 (PY5018), Mlc1·GFP prenylated expressing Scarlet-Sec4 (PY5831), control expressing GFP-Sec4 (PY4809), Mlc1·Sec4-stabilized (PY5405 with GFP-Sec4), were grown and imaged as described above for panel A. The images at the top of panel A show examples of sum projection of filament tips (bar, 2.5 μm) (the two examples for Mlc1·Sec4-stabilized strain illustrate the variation of Spitzenkörper signals). Vesicle clusters were identified in sum projections by signal intensities 8 standard deviations above the mean for Scarlet-Sec4 and 13 standard deviations above the mean for GFP-Sec4. Intensity values were normalized to the means of the control strains (*mlc1Δ*/*MLC1* expressing Scarlet-Sec4, PY5018 for the Mlc1·GFP prenylated strain, and wild-type expressing GFP-Sec4, PY4809 for the Mlc1·Sec4-stabilized strain). Values are means (horizontal lines) ± standard deviations (error bars) (*n *= 54), with *** and **** indicating *P* values of 0.0004 and <0.0001, respectively.

**FIG 7:**
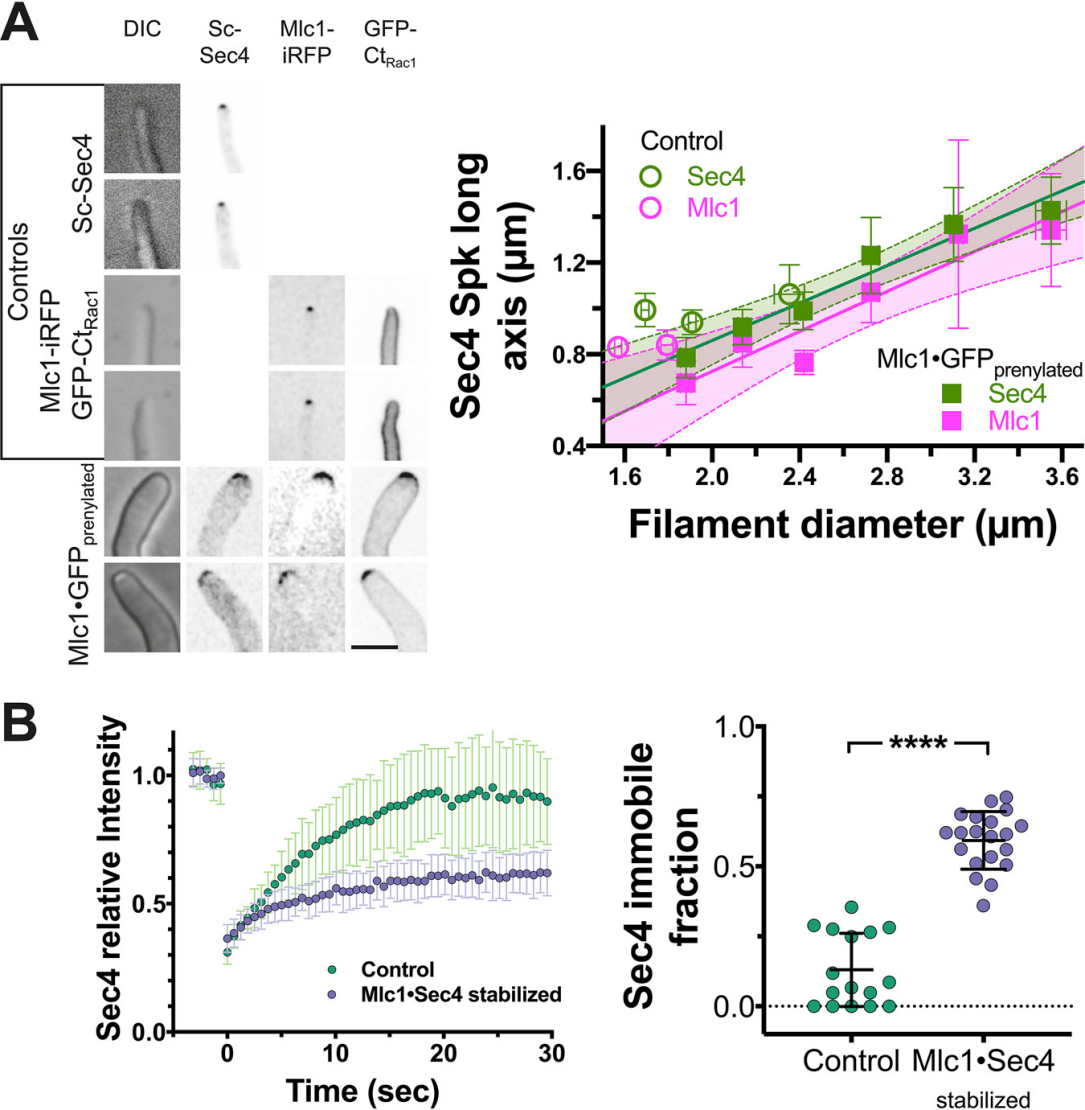
Synthetic physical interaction of Mlc1 alters the distribution of secretory vesicles and immobile fraction. (A) Secretory vesicle clusters are more spread out in Mlc1·GFP prenylated strain. Images (sum projections) of control (*mlc1Δ*/*MLC1*; PY5018) and Mlc1·GFP prenylated (Mlc1-iRFP-GNB and GFP-Ct_Rac1_; PY5831) strains expressing Scarlet-Sec4, as well as the control strain expressing Mlc1-iRFP and GFP-Ct_Rac1_, PY4776, with examples of wider filaments shown (left). Sum projections of 41 × 0.2 μm z-sections (left). The graph shows the quantitation of Scarlet-Sec4 and Mlc1-iRFP long axis in respective strains (PY5018, PY4776, and PY5831) (right) with filament diameter. Filament diameter and Mlc1 or Sec4 long axis were determined from sum projections of 50 to 70 cells, and values were binned every 0.3-μm filament diameter (6 to 26 cells per bin) with mean values and standard error of the mean shown. The lines fit for Sec4 or Mlc1 have an *r*^2^ of 0.95 or 0.90; note that the *y*-intercepts are very close to 0 with 95% confidence level prediction shown in light green (Sec4) or magenta (Mlc1). Bar, 5 μm. (B) Stabilization of Mlc1-Sec4 interaction at the Spitzenkörper increases the Sec4 immobile fraction. Fluorescence recovery after photobleaching of GFP-Sec4 in the indicated strains (Control; wild-type expressing GFP [PY4809] and Mlc1·Sec4 stabilized expressing Mlc1-iRFP-GNB and GFP-Sec4 [PY5405]). (Left) The mean FRAP recovery curve (*n *= 16 to 20 cells) with standard deviation shown (left panel). An increase in the immobile fraction is observed in the stabilized strain (right panel). Immobile fraction values with standard deviations are shown; **** indicates a *P* value of <0.0001.

We next examined the cluster of Sec4 present at the filament tips of all three *mlc1* mutants. Both the *mlc1* deletion mutant and the Mlc1·Sec4-stabilized mutant had increased Sec4 signal compared to control strains (Fig. 6A and B), suggesting an increased number of secretory vesicles. In contrast, there was a reduction in Sec4 signal in the Mlc1·GFP prenylated mutant (Fig. 6B), and the length of the vesicle cluster long axis, visualized by Sec4 and Mlc1, increased as a function of filament diameter (Fig. 7A), consistent with a more spread-out distribution of secretory vesicles. We next examined the dynamics of Sec4 at the Spitzenkörper, using fluorescence recovery after photobleaching (FRAP). We observed a striking increase in the Sec4 immobile fraction in the Mlc1·Sec4-stabilized mutant (Fig. 7B), but not in the *mlc1* deletion mutant or the Mlc1·GFP prenylated mutant compared to their respective controls (Fig. 3A and B). We confirmed that the increase in Sec4 immobile fraction observed in the Mlc1·Sec4-stabilized mutant was not due to a difference in the fraction of the total Sec4 bleached between this strain and a control strain (Fig. 3C). This Sec4 immobile fraction in the Mlc1·Sec4-stabilized mutant is similar to that previously observed for Mlc1 in wild-type hyphal filaments (10). We did not observe a significant difference in the Sec4 FRAP half-life (*t*_1/2_) values in the different mutants, although it was slightly lower in the Mlc1·GFP prenylated mutant (Fig. 3A and B). As is the case with mammalian Rab1 (41), GDP dissociation inhibitor (GDI)-mediated recycling is likely to account for the recovery of Sec4 signal after photobleaching, which is substantially faster (10) than the turnover of vesicles at the Spitzenkörper (16). Together, these results indicate that a synthetic interaction between Sec4 and Mlc1 stabilizes the association between these two proteins and suggest that targeting Mlc1 to the plasma membrane results in a more spread-out Spitzenkörper.

**FIG 8:**
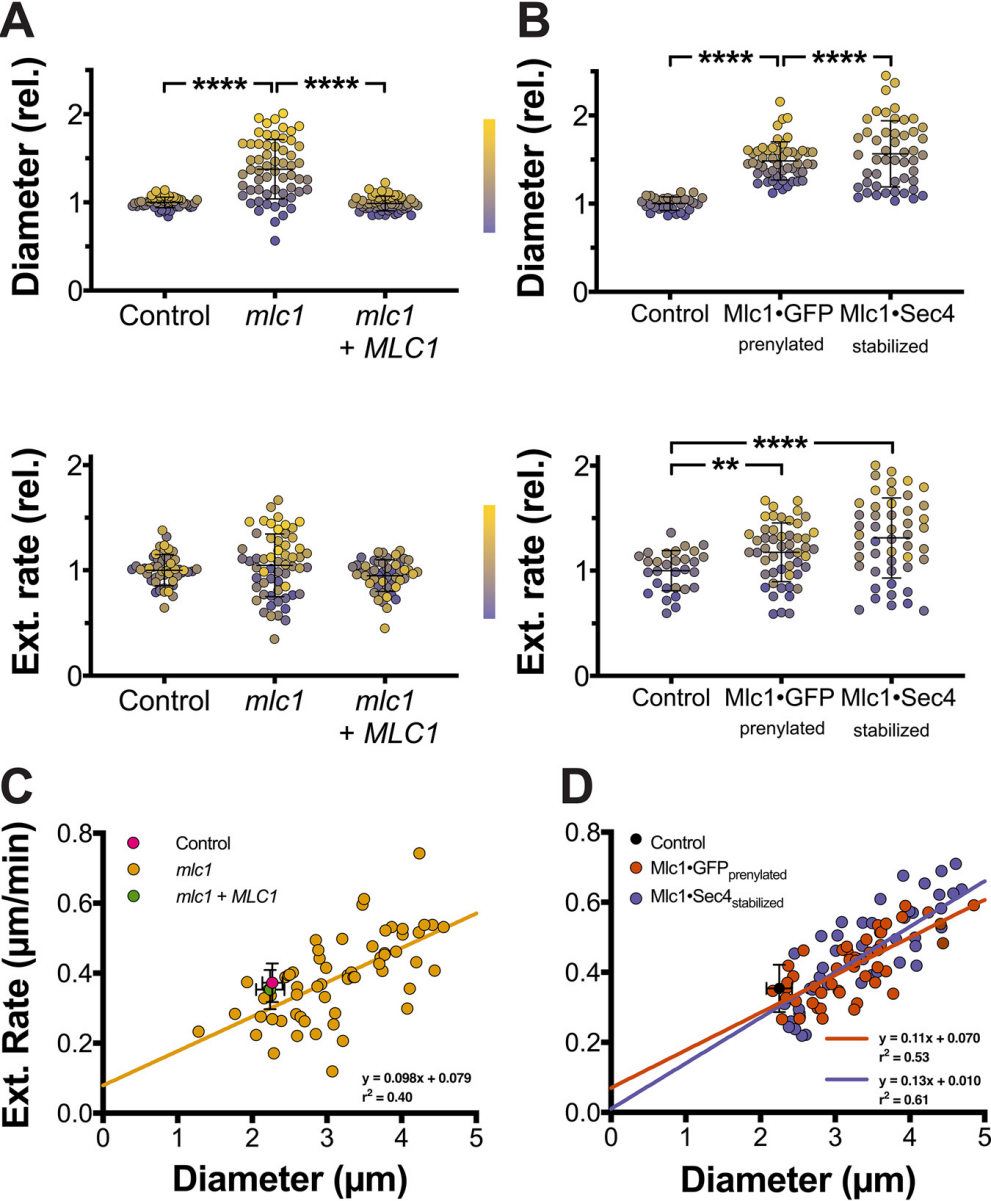
The Spitzenkörper is a critical regulator of filamentous growth. (A) The myosin light chain is required for maintaining filament diameter and extension rate. Filament diameters (top) and extension rates (bottom) were determined from time-lapse experiments as described in the legend to Fig. 1A. Diameters are the averages of values measured every 5 to 10 min over a 120-min time-lapse experiment, and extension rates are from linear fits of filament length over at least 60 min (*r*^2^ > 0.9). Diameters were normalized to the control strain *mlc1Δ/MLC1*, which had a mean value of 2.3 ± 0.1 μm. Extension rates were normalized to the control strain *mlc1Δ/MLC1* mean value of 0.37 ± 0.06 μm/min. Values were sorted by filament diameter and color coded with a color gradient from purple (smallest diameter) to yellow (largest diameter) for each strain (Lookup Table [LUT], center). Values are means (horizontal lines) ± standard deviations (error bars) (*n *= 50 to 60 cells), with **** indicating a *P* value of <0.0001. (B) Perturbation of Mlc1 distribution or stability dramatically increases growth rate. (A and B) Diameters (top) and extension (Ext.) rates (bottom) were quantitated and represented as described in the legend to Fig. 4A. They were normalized to the mean values of the wild-type control strain, 2.2 ± 0.2 μm and 0.35 ± 0.07 μm/min, respectively. Values are means (horizontal lines) ± standard deviations (error bars) (*n* = 30 to 50 cells), with ** and **** indicating *P* values of <0.005 and <0.0001, respectively. (C) In the absence of Mlc1, there is direct correlation between filament diameter and extension rate. Values from Fig. 4A were plotted with means and standard deviations for the two control strains (magenta and green circles) and values for each *mlc1* cell are shown in yellow. (D) Perturbation of Mlc1 distribution or stability also results in a direct correlation between filament diameter and extension rate. Values from Fig. 4B were plotted with the mean and standard deviation for the control strain (black circle) and values for each Mlc1·GFP prenylated or Mlc1·Sec4 -stabilized cell in red and blue, respectively.

**FIG 9:**
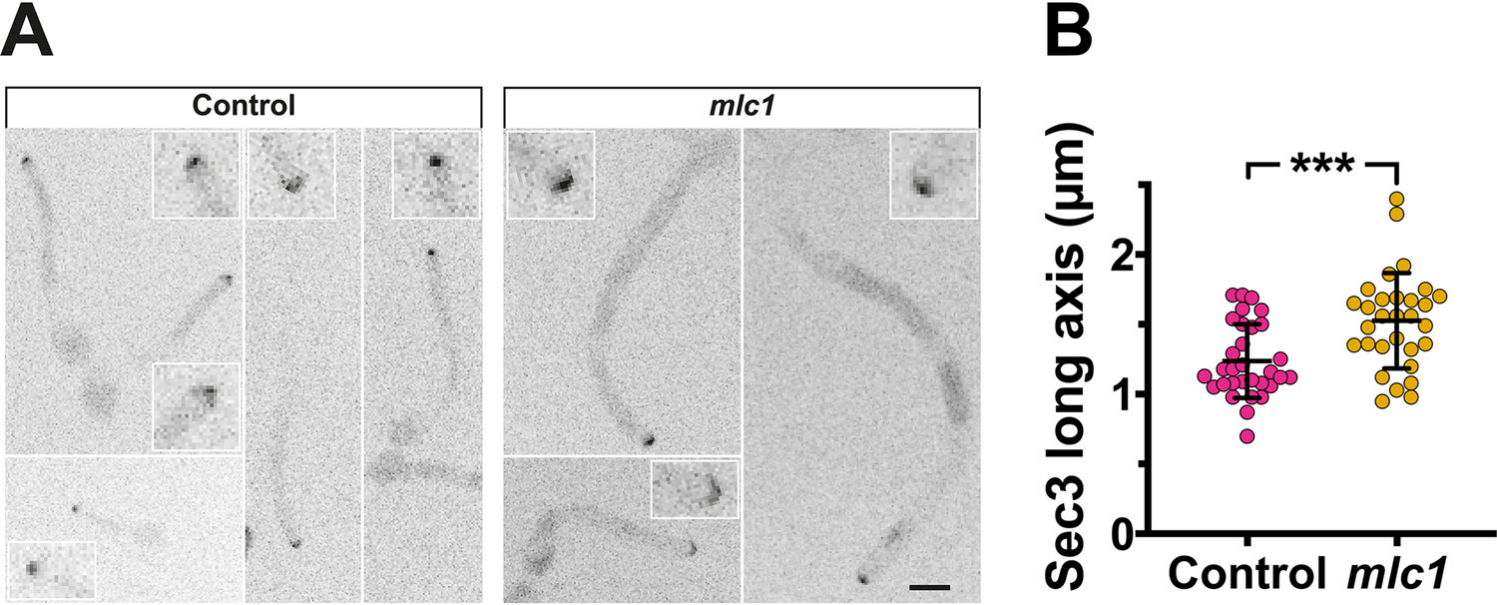
The exocyst subunit Sec3 is more spread out in the *mlc1* deletion mutant. (A) The Sec3 exocyst subunit localizes to the tip of the *mlc1* mutant. Indicated strains *mlc1Δ*/*MLC1* (Control) and *mlc1Δ*/*mlc1Δ* (*mlc1*) expressing Sec3-GFP and Scarlet-Sec4 (PY5917 and PY6007) were imaged as described in the legend to Fig. 1A with RFP images taken every 10 min and GFP images taken every 20 min. Maximum projections of 10 × 0.5 μm z-sections are showing a zoom in of the filament tips. Bar, 5 μm. (B) Sec3 is more broadly distributed in the *mlc1* mutant. The Sec3 clusters from images in panel A were identified (2.8 standard deviations above the mean signal) and long axis determined (*n *= 30), with means (horizontal lines) and standard deviations (error bars) indicated. *** indicates a *P* value of 0.005. The average extension rates were 0.25 ± 0.04 μm/min and 0.38 ± 0.09 μm/min for control and *mlc1* cells, respectively. The average filament diameters were 2.0 ± 0.2 μm and 2.8 ± 0.5 μm for control and *mlc1* cells, respectively.

Our initial examination of the three *mlc1* mutants (Fig. 3 and 5) showed that the filaments had morphological defects, and hence, we measured their diameters and extension rates from time-lapse microscopy. In all mutants, the filament diameters (Fig. S4A to D) and extension rates were constant over time. The filaments of all three mutants exhibited increased diameters, with mean diameters significantly increased compared to that of the respective control strains (Fig. 8A and B); note that the coefficient of variation was 2 to 3 times higher compared to the control strains. Similarly, both the Mlc1·GFP prenylated and Mlc1·Sec4-stabilized mutants exhibited a significant increase in the average extension rate compared to the control strains (Fig. 8A and B), and while such an increase was not apparent in the *mlc1* mutant, the coefficient of variation was increased in all three mutants. These data, color coded according to a purple-to-yellow gradient representing an increase in filament diameter, indicate that the *mlc1* mutants with larger diameters extend the fastest. This is further illustrated by the strong correlation between the filament diameter and extension rate (Fig. 8C and D), consistent with the vesicle supply center model (5), specifically $D=2\pi \frac{N}{V}$ (equation 2) predicts that when $\frac{N}{V}$ increases, so does the hyphal diameter. In the three *mlc1* mutants, there was an average of two- to fivefold increase in the rate of cell volume change (Fig. S4E and F). These results indicate that Mlc1 is critical for growth robustness. Our results are also consistent with an increased region of vesicle fusion with the plasma membrane in the *mlc1* mutant. Indeed, we examined the distribution of the key exocyst component Sec3 (34), and Fig. 9A and B show that Sec3 was found at the filament tip in the *mlc1* mutant, similar to the control strain, but with a wider distribution in wider cells (average diameter of control cells was 2.0 ± 0.2 μm compared to 2.8 ± 0.5 μm for *mlc1* mutant cells). The Sec3 signal at the filament tip was also increased in the *mlc1* mutant (normalized Sec3 signal of 1.0 ± 0.4 in wild-type cells compared to 3.2 ± 1.3 for *mlc1* mutant cells; *P* < 0.0001).

10.1128/mBio.02528-21.3 FIG S3 Sec4 dynamics at the Spitzenkörper are largely unaffected in the absence of Mlc1 and upon targeting of Mlc1 to the plasma membrane. (A) Mlc1 is not critical for Sec4 dynamics at the Spitzenkörper. Fluorescence recovery after photobleaching of Scarlet-Sec4 in Control (*mlc1Δ*/*MLC1*, PY5018) and *mlc1* (*mlc1Δ*/*mlc1Δ*, PY5451) cells; mean recovery curve (*n *= 9 to 14 cells) with standard deviation shown (left). The immobile fraction was determined as described in the legend to Fig. 7B with no significant difference between the two strains (right). (B) Targeting Mlc1 to the plasma membrane results in a slightly slower Sec4 FRAP *t*_1/2_. FRAP of Scarlet-Sec4 in indicated strains Control (wild-type; PY5433) and Mlc1·GFP prenylated (Mlc1-iRFP-GNB GFP-Ct_Rac1_; PY5831) expressing Scarlet-Sec4 with mean FRAP recovery curve (*n *= 17 or 18 cells) with standard deviation shown (left). Immobile fraction determined as described in the legend to Fig. 7B with no significant difference between the two strains for the *t*_1/2_ and immobile fraction (right). (C) The fraction of signal bleached is similar for each fluorescent protein pair. Fraction bleached is the total cell signal immediately after the bleach pulse divided by the total cell signal prior to the bleach. Values are the means of six cells with standard deviations shown with no significant difference between each pair. Download FIG S3, PDF file, 0.2 MB. Copyright © 2021 Puerner et al. 2021 Puerner et al. https://creativecommons.org/licenses/by/4.0/ This content is distributed under the terms of the Creative Commons Attribution 4.0 International license.

10.1128/mBio.02528-21.4 FIG S4 Filament diameter is constant over time in *mlc1* mutants. (A and B) Filament diameter over time. Filament diameters of indicated strains, (A) *mlc1Δ*/*MLC1* (Control; PY5018), *mlc1Δ*/*mlc1Δ* (*mlc1*; PY5451), and *mlc1Δ*/*mlc1Δ + MLC1* (*mlc1 + MLC1*; PY5661) strains, each expressing Scarlet-Sec4; (B) of wild-type strains expressing GFP-Sec4 and Mlc1-iRFP (Control, PY4809), Mlc1-iRFP-GNB and GFP-Ct_Rac1_ (Mlc1·GFP prenylated; PY5409) and Mlc1-iRFP-GNB and GFP-Sec4 (Mlc1·Sec4-stabilized; PY5405) were determined over 2-h time lapse (*n *= 25 to 50), as described in the legend to Fig. 8A. Values were spread out by cell number (*n*) − 1 for increased visibility. (C and D) Filament diameter does not change significantly over time. The slopes from panels A and B, respectively, were determined with bar indicating means and standard deviations, with no significant difference between the strains. (E and F) Filament volume increases dramatically in *mlc1* mutants. Rates of volume change were calculated from measurements in Fig. 8A and B as described in the legend to Fig. S2 and normalized to the means of the respective control strains. Values were sorted by filament diameter and color coded with a color gradient from purple (smallest diameter) to yellow (largest diameter) for each strain (LUT, right). Bars indicate means and standard deviations (*n *= 50 to 60 cells) with **** indicating a *P* value of <0.0001. Download FIG S4, PDF file, 0.6 MB. Copyright © 2021 Puerner et al. 2021 Puerner et al. https://creativecommons.org/licenses/by/4.0/ This content is distributed under the terms of the Creative Commons Attribution 4.0 International license.

**FIG 10:**
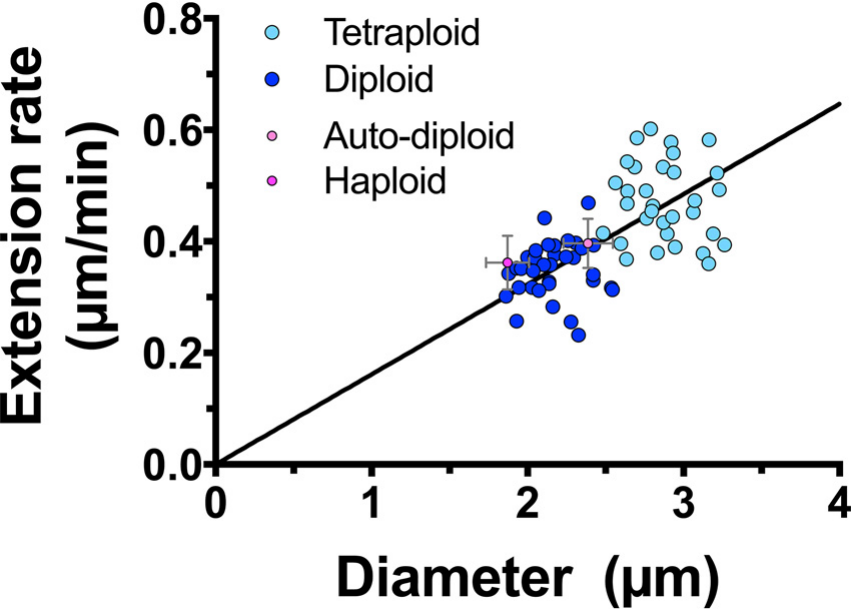
Filament diameter and extension rate are directly correlated upon changes in ploidy. Filament diameters and extension rates were determined from time-lapse experiments as described in the legend to Fig. 1A. Diameters are the averages of values measured every 10 min over a 1- to 2-h time-lapse experiment, and extension rates are from linear fits of filament length over at least 1 h (*r*^2^ > 0.95). Values for isogenic diploid (dark blue;YJB-T176) and tetraploid (light blue;YJB-T178) are shown with the means for haploid (dark magenta; PY5938) and auto-diploids (light magenta;PY5951) standard deviations, and values for each *mlc1* cell are shown in yellow (*n *= 30 to 34). A linear regression with the *y*-intercept constrained to zero yielded a *r*^2^ value of 0.36.

These results suggest that the Spitzenkörper regulates filament growth rate and morphology and that the filament extension rate is tightly linked to diameter. To investigate further this relationship, we examined C. albicans of different ploidy, as cell size has been shown to increase with increased ploidy (42). We compared two isogenic strains, and Fig. 10 shows a striking correlation between filament diameter and extension rate, both of which increase upon increased ploidy. These results further confirm that filament diameter and extension rate are linked and support the vesicle supply center model for hyphal growth.

## DISCUSSION

Our results indicate that the Spitzenkörper plays a key role in regulating hyphal growth and morphology by linking these two processes. In particular, when Mlc1 interacts with prenylated GFP, we observed increased filament extension rates and diameters. In this Mlc1·GFP prenylated mutant, the vesicle cluster is more spread out, and exocytosis is likely to be targeted to a larger area. There is precedence for such a phenotype in C. albicans, as germ tubes of the Ras-like GTPase Rsr1 deletion mutant grow faster, are somewhat wider than wild-type cells, and have an increased Mlc1 signal at the Spitzenkörper (43, 44). Intriguingly, in the Mlc1·Sec4-stabilized strain, we also observed increased filament extension rates and diameters, and we speculate that stabilizing the Sec4-Mlc1 interaction increases the targeting of vesicles to the plasma membrane. In contrast to the two SPI mutants, the average extension rate of the *mlc1* deletion mutant is not significantly increased compared to control strains, yet we observe an increased variation in the extension rate among cells, and filaments that extend faster are also wider. We speculate that this could be due to the removal of Mlc1 negative regulation on the myosin V Myo2.

Mutations in components of the Spitzenkörper alter filamentous growth. For example, deletion of C. albicans *BNI1* (33, 36), N. crassa and A. nidulans myosin V (21, 22) and A. nidulans Rab8 (12) all resulted in reduced filament extension rates. In contrast, the C. albicans *mlc1* deletion mutant extends faster than wild-type controls, consistent with this myosin light chain being a negative regulator of myosin V activity. As a Spitzenkörper was not observed in the C. albicans *bni1* (this study) and N. crassa *Δmyo-5* deletion mutants (21, 24), this suggests that this structure is critical for or associated with efficient hyphal growth.

Studies of a number of fungi indicated that as a particular fungal species grows faster, its diameter also increases. This has been observed in Ashbya gossypii (45), where filament extension rates increase over time, concomitant with an increase in filament diameter and a change in Spitzenkörper shape. Similarly, an increase in C. albicans extension rates was observed between undifferentiated and differentiated mycelia concomitant with an increase in hyphal diameter (46). In addition, an increase in N. crassa hyphal extension rate was observed going from secondary to primary branches up to leading hyphae, and this correlated with an increase in diameter (47). Most strikingly, López-Franco et al. compared a range of fungi and oomyces, including N. crassa, Gilbertella persicaria, Pythium aphanidermatum, Trichoderma viride, Saprolegnia ferax, Fusarium culmorum, and R. solani, in which an increase in hyphal diameter (from 6.7 to 11.5 μm) appears to correlate with hyphal extension rate (from 0.1 to 0.7 μm/s) (48). This relationship between hyphal diameter and extension rate has been predicted by the vesicle supply center model (equation 2) (5), and our results establish that this relationship holds upon specific perturbation of the Spitzenkörper. Interestingly, it was recently shown that variation in hyphal width and extension rate increases in faster and wider growing species (49), suggesting that the regulation of growth may be less precise when growth speeds increase. Together, our results reveal that the Spitzenkörper component Mlc1 links hyphal growth and morphology, suggesting that this structure is critical for minimizing growth and morphology variation in a fungal population, which is likely to be important for mycelium development.

## MATERIALS AND METHODS

### Strains, media, and genetic methods.

Standard methods were used for C. albicans cell culture and molecular and genetic manipulations as described previously (50). Derivatives of the BWP17 strain (51) were used in this study and are listed in Table S1 in the supplemental material. Strains were grown in rich medium (yeast extract-peptone-dextrose [YEPD]) at 30°C for all experiments, and induction of filamentous growth was carried out with fetal calf serum (FCS) at 37°C. For doxycycline gene repression, cells were grown in the presence of 20 μg/ml doxycycline (52). Oligonucleotides and synthesized DNA used in this study are listed in Tables S2 and S3. The genes encoding GFP nanobody (GNB) (53) and monomeric far-red fluorescent protein CamiRFP670 (54) were codon optimized for C. albicans and commercially synthesized (BaseClear, Netherlands). The C. albicans GNB (CaGNB) gene was cloned into pFA-GFPγ-URA3 (55) using PstI and AscI sites, resulting in pFA-CaGNB-URA3. The CamiRFP670 gene was PCR amplified and cloned into pFA-CaGNB-URA3 with unique 5′ and 3′ PstI sites (oligonucleotides CamiRFP670PstIp and CamiRFP670m-GSlink-PstI_noStop), which also introduced a GSGSGS linker between the CamiRFP670 gene and the CaGNB gene, resulting in pFA-CamiRFP670-CaGNB-URA3. The synthesized CamiRFP670 gene was PCR amplified with a unique 5′ PstI site and GAGAGA linker sequence, and a unique 3′ AscI site (CamiRFP670PstIp and CamiRFP670AscIm) and cloned into pFA-GFP-URA3 plasmid (56) to generate pFA-miRFP760-URA3. The *URA3* marker from pFA-miRFP670-URA3 was swapped for the *CdHIS1* marker from pFA-GFP-CdHIS (57) using unique AscI and PstI restriction sites.

10.1128/mBio.02528-21.5 TABLE S1 Strains used in the study. Download Table S1, DOCX file, 0.02 MB. Copyright © 2021 Puerner et al. 2021 Puerner et al. https://creativecommons.org/licenses/by/4.0/ This content is distributed under the terms of the Creative Commons Attribution 4.0 International license.

10.1128/mBio.02528-21.6 TABLE S2 Oligonucleotides used in the study. Download Table S2, DOCX file, 0.02 MB. Copyright © 2021 Puerner et al. 2021 Puerner et al. https://creativecommons.org/licenses/by/4.0/ This content is distributed under the terms of the Creative Commons Attribution 4.0 International license.

10.1128/mBio.02528-21.7 TABLE S3 Synthesized DNA used in the study. Download Table S3, DOCX file, 0.01 MB. Copyright © 2021 Puerner et al. 2021 Puerner et al. https://creativecommons.org/licenses/by/4.0/ This content is distributed under the terms of the Creative Commons Attribution 4.0 International license.

The Mlc1-miRFP670, Mlc1-miRFP670-GNB, Cdc10-GFPγ, Nop1-GFPγ, and Sec3-3xGFP strains were generated by homologous recombination, using pFA-miRFP670-CdHIS1, pFA-3xGFPγ-HIS1 (58), pFA-GFPγ-ARG4 (55), and pFA-miRFP670-GNB-URA3. The *URA3* gene was replaced by *SAT1* using homologous recombination (oligonucleotides CaURAexchS1 and CaURAexchS2 and template plasmid pFA-SAT1). Gene deletions were generated by homologous recombination, using pFA-CdHIS1, pFA-URA3, pFA-SAT1 (57), and pGEMHIS1 (52) and a doxycycline repressible *YPT31* strain as described previously (52).

pEXPARG-SEC4p-GFP-Sec4 and pEXPARG-YPT31-mCh-Ypt31 were constructed by using a 1-kb promoter and the open reading frame (ORF) followed by 1 kb downstream of the stop codon, using oligonucleotides with unique XhoI and NotI sites (CaSEC4pup1000XhoI/CaSEC4m1634NotI and CaYPT31pup1000XhoI/CaYPT31m1634NotI) and cloned into pEXPARG, resulting in pEXPARG-SEC4p-Sec4 and pEXPARG-YPT31-Ypt31, respectively. Site-directed mutagenesis (oligonucleotides CaSEC4_SDM_PACIp/CaSEC4_SDM_PACIm and CaYPT31_SDM_PAC1p/CaYPT31_SDM_PAC1m) were used to insert a unique PacI site 5′ of the ATG of the respective ORF and either yeGFP or mCherry (oligonucleotides yeGFP_PacIPUP11/yeGFP_PacIm and mCherry_PacIp/mCherry_PacIm) were then cloned into this site. Site-directed mutagenesis (oligonucleotides CaSEC4_SDM_PsiIp/CaSEC4_SDM_PsiIm and CaYPT31_SDM_PsiIp/CaYPT31_SDM_PsiIm) were used to remove the PacI site and stop codon, resulting in pEXPARG-SEC4p-GFP-Sec4 and pEXPARG-YPT31p-GFP-Ypt31.

pEXPARG-SEC4p-mSc-Sec4 was constructed using a 1,311-bp *SEC4* promoter with unique NotI and RsrII sites (using oligonucleotides CaSec4P1311pNotI and CaSec4PmRsrII), followed by codon-optimized mSc (15) with RsrII and AscI sites (oligonucleotides CamScarletFwdRsrII and CamScarletmAscI) and *SEC4* ORF flanked by AscI and MluI sites (oligonucleotides CaSec4pAscI and CaSec4mMluI) as described previously (15). The SEC4p-mSc-SEC4 cassette was cloned into pGEMURA (51) using unique NotI and SalI restriction sites, resulting in pGEMURA-SEC4p-mSc-Sec4. mScarlet was exchanged for GFPγ by using RsrII and AscI restriction sites (oligonucleotides GFPgpRsrII and CaGFPymAscI). These plasmids were linearized with BglII, which cuts within the *SEC4* promoter, and integrated at the *SEC4* locus. Additionally, for some strains (PY4554 and PY4709), the cassette was PCR amplified, using the primers CaSec4KIpURAmScar and CaSec4m1139 and then transformed into cells to replace the genomic *SEC4* copy with a fluorescently tagged copy. The *MLC1* gene with 1 kb upstream and downstream (3,479 bp) was PCR amplified from genomic DNA (gDNA) (oligonucleotides MLC1pup1000XhoI and MLC1m2479NotI), with unique 5′ XhoI and 3′ NotI restriction sites and replaced pARF3ARF3 in pEApARF3ARF3 (59), resulting in pEA-MLC1p-MLC1-MLC1t plasmid. The sequences of the *SEC4* and *MLC1* ORFs were confirmed by sequencing. pExpARG plasmids were digested with StuI for integration at the *RP10* locus.

### Microscopy and image analysis.

Cells were imaged as described previously using spinning-disk confocal microscopy (15, 60–62) with a PLANAPO total internal reflection fluorescence (TIRF) 1.45-numerical-aperture (NA) 100× objective. Exponentially growing cells were spotted on YEPD agar pads at 30°C or mixed with an equal volume of FCS and spotted on 25% (vol/vol) YEPD agar containing 75% (vol/vol) FCS pads at 37°C (15, 60). Typically, cells were incubated on the pads for 30 min prior to microscopy. Bars in all images are 5 μm. Statistical significance was determined with Student’s unpaired two-tailed *t* test (GraphPad Prism v.8-4).

Quantification of the number of secretory vesicles in the Spitzenkörper using mScarlet-Sec4-expressing strains was carried out with three-dimensional images (0.2-μm z-sections) from intensity values of live imaged filaments (10 to 20 μm long). Z-stacks were deconvolved using Huygens Professional software (v.18.04; SVI, Netherlands), with a signal-to-noise ratio of 10, and images were analyzed using Volocity software version 6.3 (Perkin Elmer USA). Single particles and clusters of mScarlet-Sec4 signals were identified using a signal intensity of 5.5 standard deviations above the mean. An average intensity for single particles of Sec4 was calculated as the average of 2 to 8 voxel objects (0.0068 to 0.027 μm^3^). The intensity of the Spitzenkörper cluster was divided by the average single vesicle intensity to estimate the number of vesicles in the identified Spitzenkörper cluster. For the analyses of Sec4 Spitzenkörper intensity and long axis, images were acquired as described above, and Volocity software was used to quantitate z-stack sum projections. Spitzenkörpers were identified by different numbers of standard deviations above the mean signal intensity, 8 for mScarlet-Sec4 and 25 for GFP-Sec4.

Budding cell doubling time was determined from time-lapse microscopy, from bud emergence to bud emergence. The aspect ratio and area of budding cells were determined by fitting an ellipse region of interest (ROI) to cell maximum projection (0.4-μm z-steps), using FIJI to extract area and aspect ratio. Filamentous cell diameter and extension rates were determined by measuring the cell length and diameter at each time point (every 5 to 10 min) during a 120-min time lapse. The extension rate was determined as previously described (62). Briefly, length was plotted over time, and the slope of a best-fit line was used as the extension rate. Cell diameter was also measured at each time point at the center of the hyphal filament, and the numbers reported are the average over the time course. Volume was calculated at each time point using the cylindrical volume formula:

$$V=\text{π}r^{\text{2}}h$$

where *r* is the radius and *h* is the cell length. Change in volume was determined by plotting volume over time and using the slope of a best-fit line.

Fluorescence recovery after photobleaching (FRAP) analysis was performed as described previously (15). Images were captured every 0.63 s at 0.7% or 0.2% maximum laser intensity for 488-nm or 561-nm laser lines, respectively. Photobleaching scans on a circular area of 1 to 2 μm^2^ were carried out with five consecutive pulses at 80% or 50% laser intensity for 488-nm or 561-nm laser lines, respectively, using a 1.63-μs pixel dwell time. The average signal intensity of the bleach ROI was normalized to photobleaching during image acquisition, which was fit to a one-phase decay regression: *Y* = (*Y*_0_ − plateau)(*e*^−^*^kx^*) + plateau, of the average intensity elsewhere in the cell (using GraphPad Prism 8 software). Regression analysis to determine the FRAP *t*_1/2_ was done using a one-phase exponential association function in GraphPad Prism 8 software as follows: *Y* = *Y*_max_(1 − *e*^−^*^kx^*), where *k* is the rate constant and *t*_1/2_ is 0.69/*k*. The immobile fraction was calculated using the equation, 1 – [(*I*_final_ − *I*_postbleach_)/(*I*_prebleach_ − *I*_postbleach_)], where *I* is the signal intensity.
