# Supplementary figures and images for: A Myosin Light Chain Is Critical for Fungal Growth Robustness in Candida albicans
Source: mBio. 2021 Oct 5;12(5):e02528-21. doi: 10.1128/mBio.02528-21 (PMC8546852; doi:10.1128/mBio.02528-21)

**A**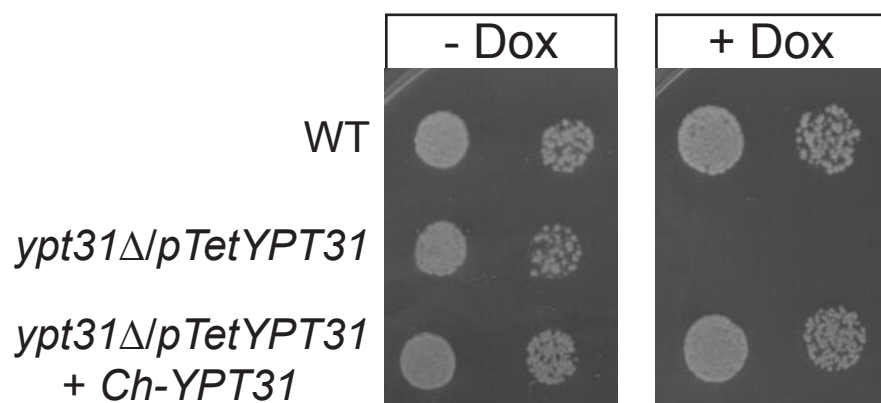**B**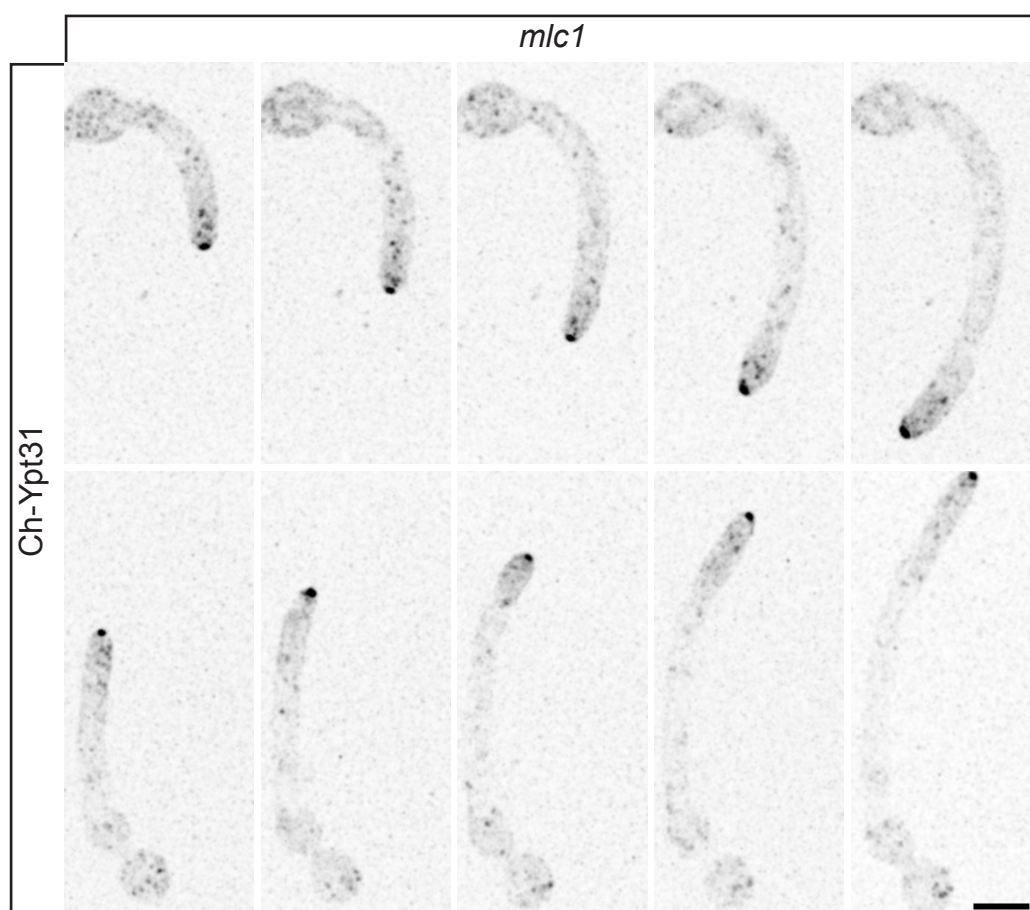

Figure S1

Supplement: FIG S1 [file mbio.02528-21-sf001.pdf]

**A**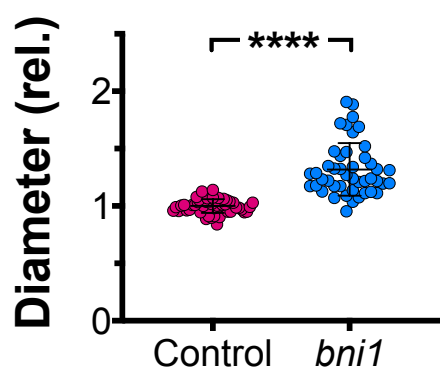**B**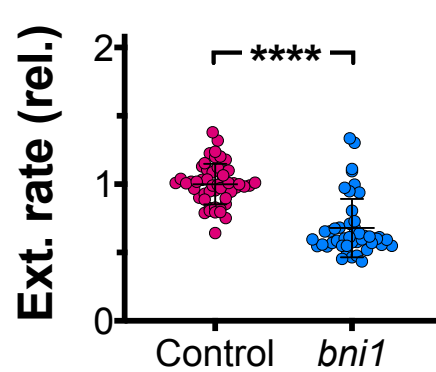**C**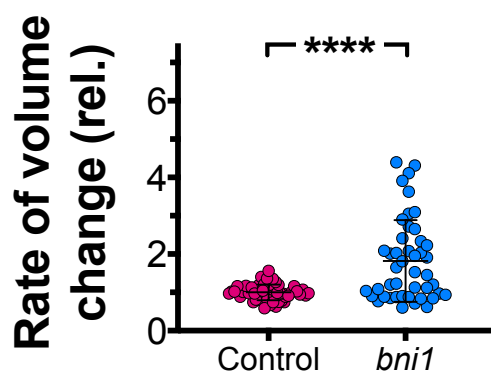**D**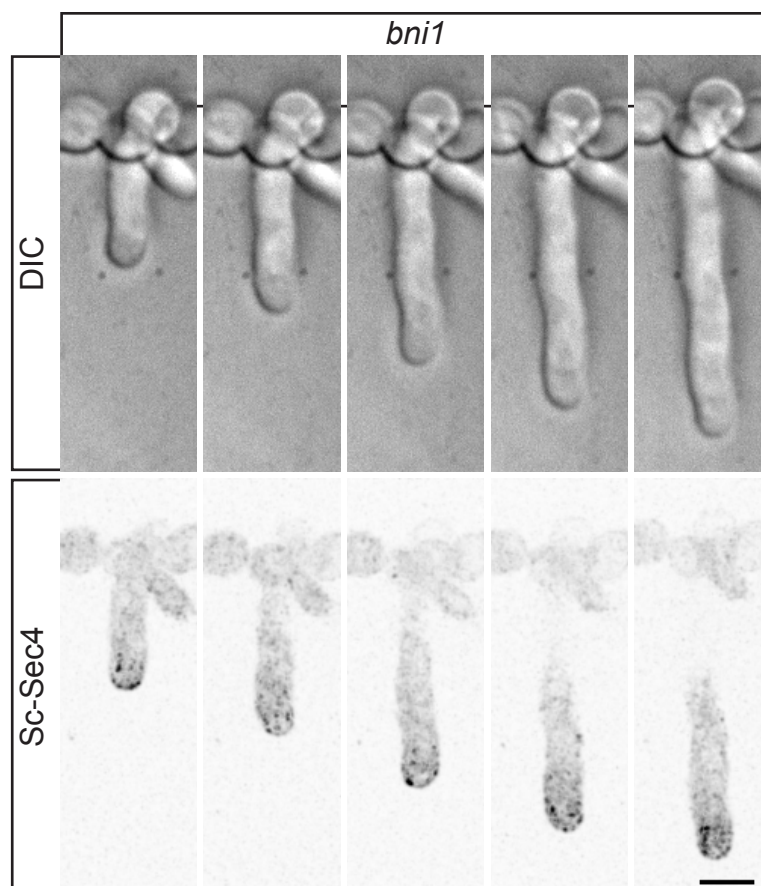

Figure S2

Supplement: FIG S2 [file mbio.02528-21-sf002.pdf]

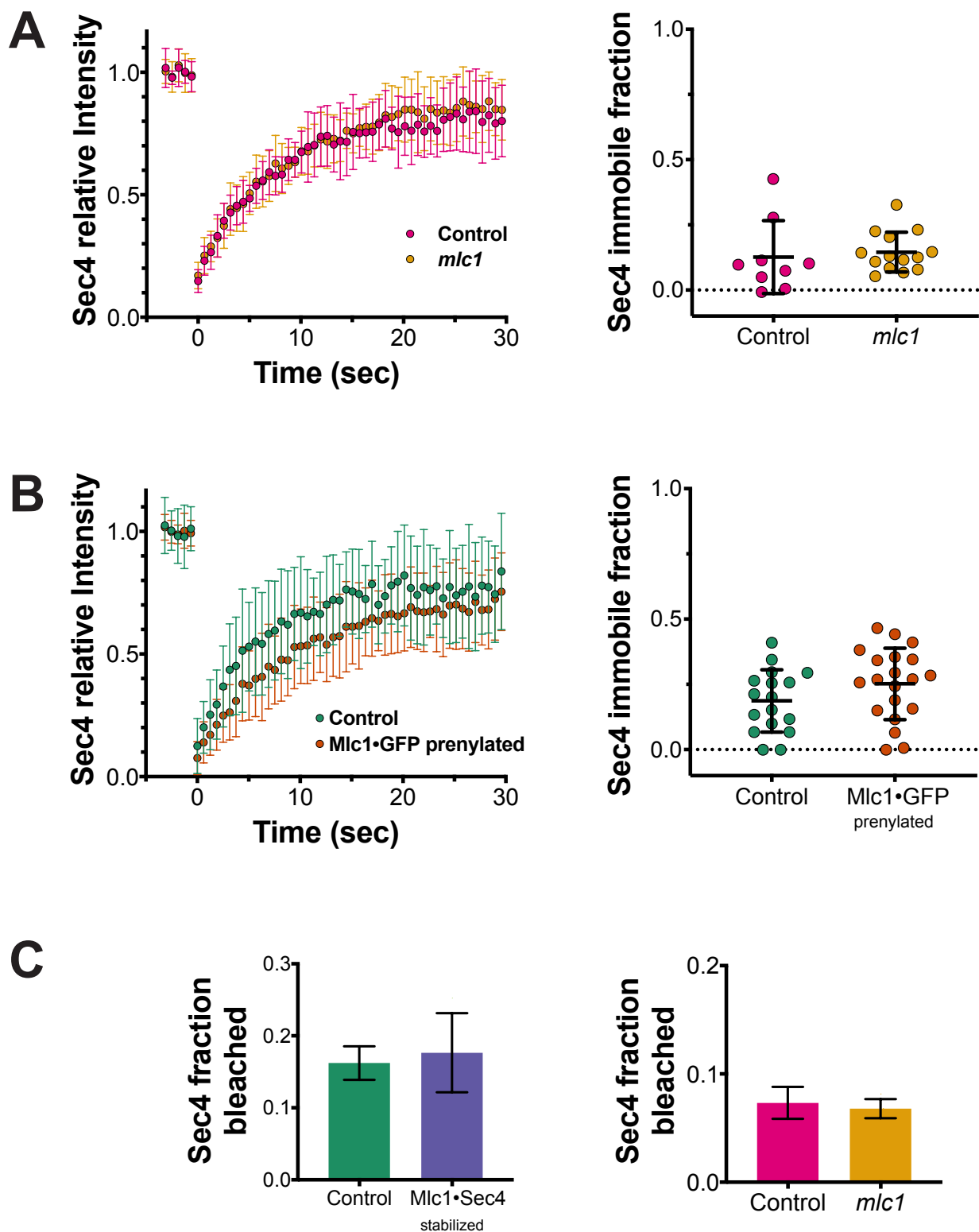

Figure S3

Supplement: FIG S3 [file mbio.02528-21-sf003.pdf]

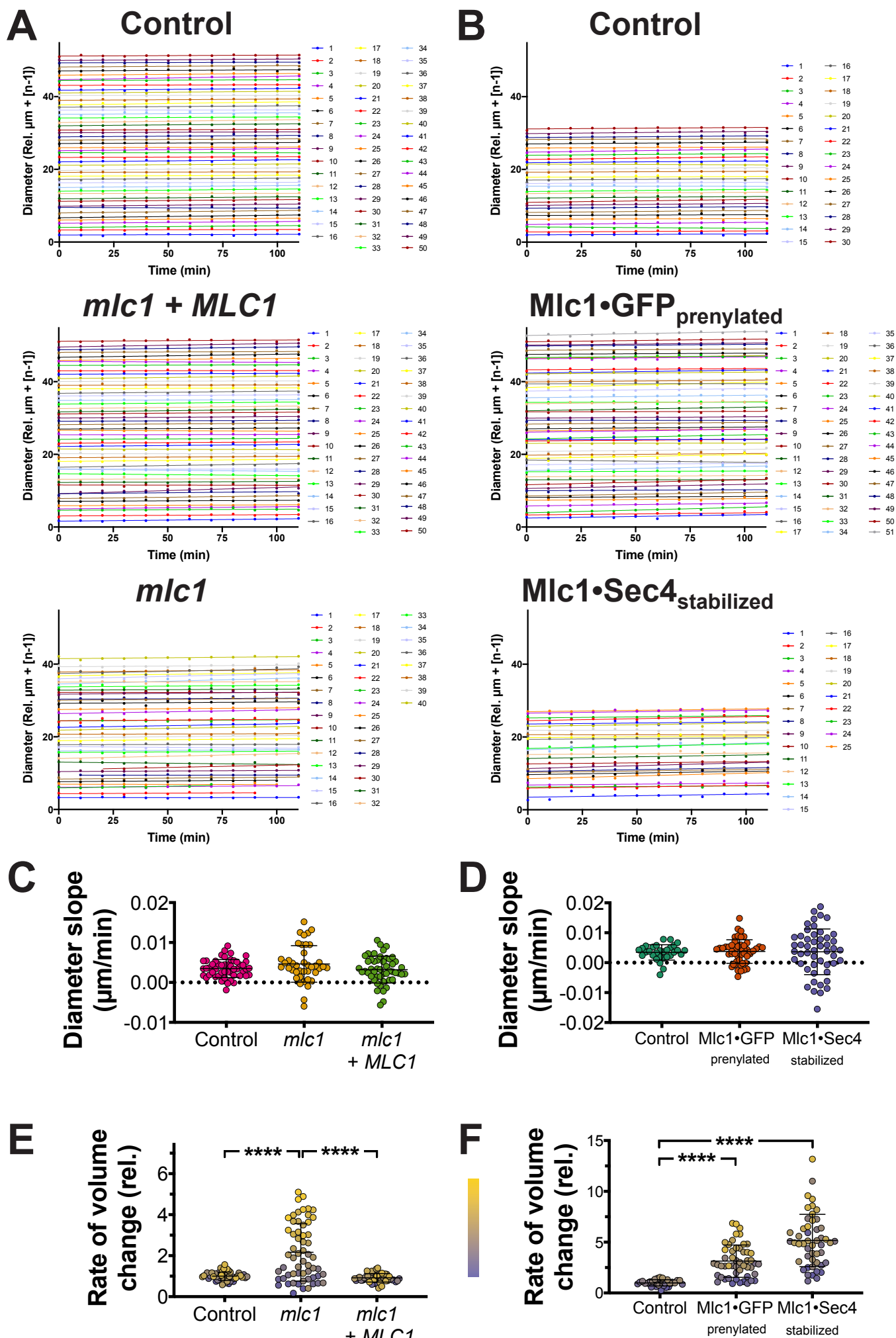

Figure S4

Supplement: FIG S4 [file mbio.02528-21-sf004.pdf]
