# Supplementary material for: A Myosin Light Chain Is Critical for Fungal Growth Robustness in Candida albicans
Source: mBio. 2021 Oct 5;12(5):e02528-21. doi: 10.1128/mBio.02528-21 (PMC8546852; doi:10.1128/mBio.02528-21)
Supplement: TABLE S1 [file mbio.02528-21-st001.docx]

**Supplemental Table 1: Strains used in the study.**

| STRAIN | RELEVANT GENEOTYPE | REFERENCE |
| --- | --- | --- |
| BWP17 | *ura3Δ::λimm434/ura3Δ::λimm434 his1Δ::hisG/his1Δ::his arg4::hisG/arg4Δ::hisG* | (51) |
| YJB-T900 | Haploid; *MTL****a*** *ura3Δ::imm434 his4 gal1Δ::ura3Δ::HIS4* | (64) |
| YJB-T1655 | Auto-diploid of YJB-T 900 | (64) |
| YJB-T176 | Diploid Mat**a**; *MTL****a****/MTLα1∆α2∆ gal1::hisG/gal1::hisG ura3- ENO1/ENO1::RFP-SAT1* | (42) |
| YJB-T178 | Tetraploid Mat**a/**α; *MTL****a****/MTL****a****∆ MTLα/MTLα1∆α2∆ gal1::hisG/gal1::hisG/∆/gal1::hisG/gal1::hisG URA3/ura3 ENO1/ENO1::RFP-SAT1/ENO1/ENO1::RFP-SAT1* | (42) |
| WYL2 | Same as BWP17 with *bni1Δ::ARG4/bni1Δ::HIS1* | (36) |
| PY173 | *ade2Δ::hisG/ade2Δ::hisG ura3Δ::λimm434/ura3Δ::λimm434 his1Δ::hisG/his1Δ::hisG arg4Δ::hisG/arg4Δ::hisG ENO1/eno1::ENO1-tetR ScHAP4AD- 3xHA-ADE2* | (52) |
| PY2705 | Same as PY173 with *sec4∆::HIS1/SEC4* | This study |
| PY2862 | Same as PY173 with *ypt31::URA3-pTet_off_YPT31* | This study |
| PY2896 | Same as PY2862 with *ypt31∆::HIS1* | This study |
| PY3455 | Same as PY2896 with *RP10::ARG4*-*YPT31p-mSc-YPT314* | This study |
| PY4501 | Same as BWP17 with *RP10::ARG4*-*SEC4p-GFP-SEC4* | This study |
| PY4554 | Same as PY2705 with *sec4*::*URA3-SEC4p-mSc-SEC4* | This study |
| PY4687 | Same as BWP17 with *mlc1∆::SAT1/MLC1* | This study |
| PY4709 | Same as BWP17 with *sec4*::*URA3-SEC4p-GFP-SEC4* | This study |
| PY4754 | Same as BWP17 with *mlc1∆::SAT1/mlc1∆::URA3* | This study |
| PY4809 | Same as PY4709 with *MLC1::MLC1-miRFP670-CdHIS1* | This study |
| PY5018 | Same as PY4687 with *SEC4*::*URA3*-*SEC4p*-*mSc-SEC4* | This study |
| PY5020 | Same as PY4754 with *RP10::ARG4*-*YPT31p-mCh-YPT31* | This study |
| PY5385 | Same as BWP17 with *MLC1::MLC1-miRFP670-GNB-URA3* | This study |
| PY5405 | Same as PY5385 with *RP10::ARG4*-*SEC4p-GFP-SEC4* | This study |
| PY5409 | Same as PY5385 with *RP10::ARG4*-*ADH1-GFP-CtRac1* | This study |
| PY5433 | Same as BWP17 with *SEC4*::*URA3*-*SEC4p*-*mSc-SEC4* | This study |
| PY5435 | Same as WYL2 with *SEC4*::*URA3*-*SEC4p*-*mSc-SEC4* | This study |
| PY5451 | Same as Same as BWP17 with *mlc1∆::SAT1/mlc1∆::CdHIS1*  *SEC4*::*URA3*-*SEC4p*-*mSc-SEC4* | This study |
| PY5658 | Same as PY4754 with *RP10::ARG4*-*MLC1p-MLC1* | This study |
| PY5661 | Same as PY5451 with *RP10::ARG4*-*MLC1p-MLC1* | This study |
| PY5713 | Same as PY5018 with *CDC10::CDC10-GPFγ-ARG4* | This study |
| PY5716 | Same as PY5018 with *NOP1::NOP1-GPFγ-ARG4* | This study |
| PY5717 | Same as PY5451 with *CDC10::CDC10-GPFγ-ARG4* | This study |
| PY5720 | Same as PY55451with *NOP1::NOP1-GPFγ-ARG4* | This study |
| PY5812 | Same as BWP17 with *MLC1::MLC1-miRFP670-GNB-SAT1 RP10::ARG4*-*ADH1-GFP-CtRac1* | This study |
| PY5831 | Same as PY5812 with *sec4*::*URA3-SEC4p-mSc-SEC4* | This study |
| PY5917 | Same as PY5018 with *SEC3::SEC3-3xGFP-HIS1* | This study |
| PY5938 | Same as YJB-T 900 with *ADH1::ADH1-mSc-URA3* | This study |
| PY5951 | Same as YJB-T1655 with *NEUT5L*::*SAT1*-*ADH1p*-*GFP-CtRac1* | This study |
| PY6007 | Same as BWP17 with *mlc1∆::SAT1/ mlc1∆::ARG4 SEC4*::*URA3*-*SEC4p*-*mSc-SEC4 SEC3::SEC3-3xGFP-HIS1* | This study |
