# Supplementary material for: A Myosin Light Chain Is Critical for Fungal Growth Robustness in Candida albicans
Source: mBio. 2021 Oct 5;12(5):e02528-21. doi: 10.1128/mBio.02528-21 (PMC8546852; doi:10.1128/mBio.02528-21)
Supplement: TABLE S2 [file mbio.02528-21-st002.docx]

**Supplemental Table 2: Oligonucleotides used in the study.**

| Primer | Sequence |
| --- | --- |
| CamiRFP670m-GSlink-PstI_noStop | CCTctgcaggctaccgctgccgctaccTGATTCTAAAGCAGTAATTCTAGTAGCAATTCTTTC |
| CamiRFP670PstIp | gtactgcagGGTGCTGGCGCAGGTGCTgttgctggtcatgcttcagg |
| CamiRFP670AscIm | cgcggcgcgccTTATGATTCTAAAGCAGTAATTC |
| CaSEC4pup1000XhoI | ATCTCGAGctggatagaggaagacaaag |
| CaSEC4m1634NotI | TATAGCGGCCGCttgtatagatttagttcttgtttg |
| CaSEC4_SDM_PACIp | GAAAAACATACTAAGTATTAACttaattaaATGAGCGGTAAAGGAACATCATC |
| CaSEC4_SDM_PACIm | GATGATGTTCCTTTACCGCTCATttaattaaGTTAATACTTAGTATGTTTTTC |
| CaSEC4_SDMp_PsiI | GACTACGCATTAATTAtAATGAGCGGTAAAGG |
| CaSEC4_SDMm_PsiI | CCTTTACCGCTCATTATAATTAATGCGTAGTC |
| CaYPT31m1670NotI | TATAGCGGCCGCacacacactattggtagcgc |
| CaYPT31pup1000XhoI | ATCTCGAGttagtggttgattcgggtattg |
| CaYPT31pKO | gaataaatacttaataagtgaaatacccctttttttttcaaagaaagacgtacaatataaactaatttgacattcataATGTGTGGAATTGTGAGCGGATA |
| CaYPT31mKO | ctcaggttggatccctctttcttgactagtgtttctttttttaatgtggtttttgctatttttgtcaccttcctaatgTTTCCCAGTCACGACGTT |
| CaYPT31pTetOKin | GGGAAGTAAAACTACATTGAAAAAAAAAAGAGAGACATAACTCAAAAATTTAGATTGAATAAATACTTAATAAGTGAAATACCaggaattgatttggatgg |
| CaYPT31mTetOKin | CAACTCCTGAATCCCCAATCAACACTATTTTATATAAATATTCGTAATCGTAGGAGTAATCGTCGCTGTTATCGGCCATctagttttctgagataaagctg |
| CaYPT31_SDM_PACIp | CGTACAATATAAACTAATTTGATTAATTAAATGGCCGATAACAGCGACGATTACTCC |
| CaYPT31_SDM_PACIm | GGAGTAATCGTCGCTGTTATCGGCCATTTAATTAATCAAATTAGTTTATATTGTACG |
| CaYPT31_SDMp_PsiI | GACTACGCATTAATTATAATGGCCGATAACAG |
| CaYPT31_SDMm_PsiI | CTGTTATCGGCCATTATAATTAATGCGTAGTC |
| CaSec4pAscI | aaaggcgcgccgATGAGCGGTAAAGGAACATC |
| CaSec4mMluI | atcacgcgtaCTAGCAACAGTTATTCTTTGC |
| CaSec4P1311pNotI | GTAGACGCGGCCGCaaagctgttaaattggaaaatccaaag |
| CaSec4PmRsrII | cgatcgCGGTCCGCGGACCGggatgaattgttaatacttag |
| mCherry_PacIp | gccttaattaaaATGGTTTCAAAAGGTGAAG |
| mCherry_PacIm | ggttaattaatgcgtagtctgggacatcgtatgggtaggATTTATATAATTCATCCATACCACC |
| yeGFP_PacIPUP11 | gcttaattaacagatcatatgtctaaaggtgaagaattattcac |
| yeGFP_PacIm | GGTTAATTAATGCGTAGTCTGGGACATCGTATGGGTAGGATTTGTACAATTCATCCATACCATGGG |
| CamScarletFwdRsrII | ctcgcggtccgcggaccgATGGTTTCAAAAGGTGAAG |
| CamScarletmAscI | cggcgcgccTTTATATAATTCATCCATACCACC |
| GFPgpRsrII | acgcggaccgATGTCTAAAGGTGAAGAATTATTCACTGG |
| CaGFPymAscI | cggcgcgccTTTGTACAATTCATCCATACC |
| CaSec4KIpURAmScar | GTTGGCAAGTTCTCAAGCTGGTTATTTAGAAGTCATGCCTTTGACTTTATCTTCTCACGATGAGAATTTTGCTTGGTTGAAACAGAAGGATGATGGTACCgggcccgacgtcgcatgctc |
| CaSec4m1139 | GGTGGTTATAAATGAAGGG |
| MLC1pup1000XhoI | GTCTGTctcgagTAACTGTCCAATTTGATTCTTTGGC |
| MLC1m2479NotI | ACgcggccgcACCGAACAAGCTAAAACCTTAGGTGC |
| CaMLC1pKO | CTGTCACTGATAGATTATGTAGGCTTCACTAGTTGCATTGTGCAGGGTAAACTTTTTTTTTGTCTGATCCCAAAAGAGTAGTTTGTTTACATTTTTTCTGgaagcttcgtacgctgcaggtc |
| CaMLC1mKO | GTTACTTTTCCTCTCTTACTCTCTCTCTTTTCATTGGCATATATTACTCTCCAAAGTAACTTATCAAGTACTACATAAAACTTCAAATAAACGGTATCCAATTCGtctgatatcatcgatgaattcgag |
| CaURAexchS1 | tttcccagtcacgacgttgtaaaacgacggccagtgaattgtaatacgactcactatagggcgaattggGAAGCTTCGTACGCTGCAGGTC |
| CaURAexchS2 | tgtggaattgtgagcggataacaatttcacacaggaaacagctatgaccatgattacgccaagctcggTCTGATATCATCGATGAATTCGAG |
| CaSec4pKO | cctccccgttttattttttaatttttgttcaaaatcatttcaagaaaaacatactaagtattaacaattcatccATGTGTGGAATTGTGAGCGGATA |
| CaSec4mKO | ctcttcttcttcttcttcgttcatggtaaaatataatctaacagataaagtaaaacaaacaagcttctctagcaacagTTTCCCAGTCACGACGTT |
| CaMLC1_S1miRFP670p | TATTAAAAGGGGTCAATGTAACTTCTGATGGAAATGTGGATTATGTTGAATTTGTCAAATCAATTTTAGACCAAGGTGCTGGCGCAGGTGCTGTTGCTGG |
| CaMLC1pFA | GATGAGTTATTAAAAGGGGTCAATGTAACTTCTGATGGAAATGTGGATTATGTTGAATTTGTCAAATCAATTTTAGACCAAGGTGCTGGCGCAGGTGCTTC |
| CaMLC1mFA | CGAACAAGACTATACAATAACTATAATTTGTAAAACTTGTAGTATATATATTTCAATGGTTAATTGTAAATTTTCTTTTATTCTGATATCATCGATGAATTCGAG |
| CaCdc10pFA | caatcaaaccaagatttgaagaacacctctggtgtgccaaatgctcctatgttccaatcaactacaggtactgctgctgctagaGGTGCTGGCG AGGTGCTTC |
| CaCdc10mFA | CGCGTTTTGCTTTTCAACAAACACACAAAAGAAGAGGAATACAAAAAAGTAAAATCACATTATATCAATAACAAACctgatatcatcgatgaattcgag |
| CaNop1KI_S1 | GAGAAGTCCAAAAATTGAGAGAAGAAAGAATTAAACCATTGGAACAATTGACCTTGGAACCTTATGAAAGAGACCATTGTATTGTTGTTGGTAGATACATGAGAAGCGGAATAAAGAAAggtgctggcgcaggtgcttc |
| CaNop1KI_S2 | CCAGTGTTTCCAAAATTTTCATTCATTCTTTATTTTTTTTTAGTTTTCAATAATCAAATGTATTAATCCTATTGTACAAAATATTTTTATTTAAAATTTAGAGTATCCCAAAATAACCTAATtctgatatcatcgatgaattcgag |
| caSec3longxFP_S1 | GGAAATGATATAGGGTCTGCTTTGAATGAAGTGGATAATATGACTCAGATTTTCCAGAAGATGGAGGTGAGATTGAAACTTGTACGAAATGAGCTACAAAGTTCTGCTACTGCTggtgctggcgcaggtgcttc |
| caSec3longxFP_S2 | GTTGTATATGTAGTAGAGAAAGCAGTACTAAAAACAGTATTAATTAATTAAAGCTATACTATACAAACTTAATAATTACTATACTCTTGATAAAAAGACTTGCTCCATTtctgatatcatcgatgaattcgag |
| CaADH1KIxFP_S1 | ccagaattatttttttttcatcagtttaacaacaacaaacgttattgtcatacaacaacaacaacaaatacaaaaacaattatgGGTGCTGGCGCAGGTGCT |
| CaADH1KIxFP_S2 | ctgggtaatccttgtagactaattgaccaccattggtatcaaagacaacggctttttgagtttttgggatttgttcagacatTCTGATATCATCGATGAATTCGAG |
