# Supplementary material for: A Myosin Light Chain Is Critical for Fungal Growth Robustness in Candida albicans
Source: mBio. 2021 Oct 5;12(5):e02528-21. doi: 10.1128/mBio.02528-21 (PMC8546852; doi:10.1128/mBio.02528-21)
Supplement: TABLE S3 [file mbio.02528-21-st003.docx]

**Supplemental Table 3: Synthesized DNA used in the study.**

| DNA | Sequence |
| --- | --- |
| CaGNB | ATGGCTGATGTTCAATTAGTTGAATCAGGTGGTGCTTTAGTTCAACCAGGTGGTCATTAAGATTATCATGTGCTGCTTCAGGTTTTCCAGTTAATAGATATTCAATGAGATGGTATAGACAAGCTCCAGGTAAAGAAAGAGAATGGGTTGCTGGTATGTCATCAGCTGGTGATAGATCATCATATGAAGATTCAGTTAAAGGTAGATTTACTATTTCAAGAGATGATGCTAGAAATACTGTTTATTTACAAATGAATTCATTAAAACCAGAAGATACTGCTGTTTATTATTGTAATGTTAATGTTGGTTTTGAATATTGGGGTCAAGGTACTCAAGTTACTGTTTCATCAGGTAGATAA |
| CamiRFP670 | ATGGTTGCTGGTCATGCTTCAGGTTCACCAGCTTTTGGTACTGCTTCACATTCAAATTGTGAACATGAAGAAATTCATTTAGCTGGTTCAATTCAACCACATGGTGCTTTATTAGTTGTTTCAGAACATGATCATAGAGTTATTCAAGCTTCAGCTAATGCTGCTGAATTTTTAAATTTAGGTTCAGTTTTAGGTGTTCCATTAGCTGAAATTGATGGTGATTTATTAATTAAAATTTTACCACATTTAGATCCAACTGCTGAAGGTATGCCAGTTGCTGTTAGATGTAGAATTGGTAATCCATCAACTGAATATTGTGGTTTAATGCATAGACCACCAGAAGGTGGTTTAATTATTGAATTAGAAAGAGCTGGTCCATCAATTGATTTATCAGGTACTTTAGCTCCAGCTTTAGAAAGAATTAGAACTGCTGGTTCATTAAGAGCTTTATGTGATGATACTGTTTTATTATTTCAACAATGTACTGGTTATGATAGAGTTATGGTTTATAGATTTGATGAACAAGGTCATGGTTTAGTTTTTTCAGAATGTCATGTTCCAGGTTTAGAATCATATTTTGGTAATAGATATCCATCATCAACTGTTCCACAAATGGCTAGACAATTATATGTTAGACAAAGAGTTAGAGTTTTAGTTGATGTTACTTATCAACCAGTTCCATTAGAACCAAGATTATCACCATTAACTGGTAGAGATTTAGATATGTCAGGTTGTTTTTTAAGATCAATGTCACCATGTCATTTACAATTTTTAAAAGATATGGGTGTTAGAGCTACTTTAGCTGTTTCATTAGTTGTTGGTGGTAAATTATGGGGTTTAGTTGTTTGTCATCATTATTTACCAAGATTTATTAGATTTGAATTAAGAGCTATTTGTAAAAGATTAGCTGAAAGAATTGCTACTAGAATTACTGCTTTAGAATCATAA |
